# Charge-Transfer Interactions in Organic Functional Materials

**DOI:** 10.3390/ma3084214

**Published:** 2010-08-05

**Authors:** Hsin-Chieh Lin, Bih-Yaw Jin

**Affiliations:** Department of Chemistry, Center for Theoretical Sciences, and Center for Quantum Science and Engineering, National Taiwan University, Taipei, Taiwan; E-Mail: hsin-chieh.lin@chemistry.gatech.edu

**Keywords:** charge-transfer, cyclophane, organic materials, composite-molecule, molecule-in-molecule

## Abstract

Our goal in this review is three-fold. First, we provide an overview of a number of quantum-chemical methods that can abstract charge-transfer (CT) information on the excited-state species of organic conjugated materials, which can then be exploited for the understanding and design of organic photodiodes and solar cells at the molecular level. We stress that the Composite-Molecule (CM) model is useful for evaluating the electronic excited states and excitonic couplings of the organic molecules in the solid state. We start from a simple polyene dimer as an example to illustrate how interchain separation and chain size affect the intercahin interaction and the role of the charge transfer interaction in the excited state of the polyene dimers. With the basic knowledge from analysis of the polyene system, we then study more practical organic materials such as oligophenylenevinylenes (OPV_n_), oligothiophenes (OT_n_), and oligophenylenes (OP_n_). Finally, we apply this method to address the delocalization pathway (through-bond and/or through-space) in the lowest excited state for cyclophanes by combining the charge-transfer contributions calculated on the cyclophanes and the corresponding hypothetical molecules with tethers removed. This review represents a step forward in the understanding of the nature of the charge-transfer interactions in the excited state of organic functional materials.

## 1. Introduction

Exploring new organic semiconductor materials and understanding the relationship between the molecular structure and the properties are still major challenges. Numerous experiments indicate that fine tuning and understanding the interchain interactions are required in optimizing several organic conjugated materials such as light-emitting devices [1,2,3,4,5], photovoltaic materials [6,7,8,9,10], and field-effect transistors [11,12,13,14,15] and nonlinear optical materials [16,17,18]. In the microscopic sense, it is believed that photoexcitation of an organic material may generate charge-transfer (CT) excitons in the strong interchain interaction limit which has been described as spatially indirect excitons or bound polaron pairs. Although it would be detrimental to light emission efficiency, CT excitons can be exploited for the design of photodiodes and solar cells based on blends of organic conjugated materials [19,20,21,22,23,24,25,26,27,28]. For example, it has been shown that formation of charge-transfer exciton might enhance the photovoltaic effect in polymer solar cells [29]. The reason for this is the photo-excitation process in conjugated systems can not generate free charge carriers but neutral, bound electron-hole pairs so that charge transfer might facilitate the formation of free charge carriers [29]. Intermolecular charge transfer has been known to be short distance process in the organic conjugated materials. Monte Carlo simulations of the chain packing in the PPV derivatives resulted in 3.3-4.2 Å interchain separations [30]. The study of the family of stilbenoid dimers bound to a paracyclophane core showed that new interchromophore states with strong through-space interaction appear at short interchromophore distances [31]. More recently, the studies on the admixture of the charge transfer transitions in the excited states are increasingly being examined for many molecular systems [32,33,34,35]. These results indicate the necessity of further investigation the chromophore-chromophore interactions with short interchain separation, and how the charge transfer interactions affects the electronic structure of the aggregate in the organic solid.

Historically, the terminology of CT exciton is from the analogy with the similar species sometimes formed in conventional molecular crystals [36,37]. As a matter of fact, the terminology such as Frenkel excitons and CT excitons used conventionally in the field of molecular solids to describe the nature of excitations is recently widely adopted by the conjugated polymer community. The formation of CT-excitons in conjugated materials is known by several possible mechanisms. For example, after photogeneration of a singlet excited state, the excited electron may transfer to a neighboring polymer chain [38,39,40,41]. Therefore, this process leaves a hole on the original chain and an electron on the other which are bound together by the Coulomb interaction, and this bound electron-hole pair that constitutes the spatially indirect exciton. The positive and negative species on adjacent chains may be thought of as forming positive and negative polarons, leading to the alternative description as geminate interchain bound polaron pairs. Alternatively, another plausible mechanism to generate CT-excitons is from an excited-state dimer (excimer) or a ground-state dimer (aggregation). Such a species is stable as a result of resonance contributions from Frenckel excitons and CT excitons [42]. Note that the possibility of formation of these species is dependent on the proximity of the neighboring molecules and their relative orientation [43,44,45]. In this contribution, we review some of our recently quantum-chemical studies and discuss the importance of the CT exciton in the excited states of the polyene system as well as some organic materials, including oligophenylenevinylenes (OPV_n_), oligothiophenes (OT_n_), and oligophenylenes (OP_n_) in Section 3 [46]. Section 4 focuses on understanding the delocalization pathway (through-bond and/or through-space) in the cyclophane systems by using a CT exciton [47].

Note that although there are a number of methods reported in the literature that can abstract the charge-transfer contribution of the excited states, those methods have certain limitation for obtaining the detailed charge-transfer configuration of the excited states in the molecules. For example, the charge-transfer contribution can be calculated by a detailed analysis of the excited-state wave function of a dimer via supermolecular calculations [43]. However, the supermolecular approach does not provide the charge-transfer configuration information because of the inadequacy of the basis set construction based on completely delocalized molecular orbitals obtained by the Hartree-Fock calculations. Another approach is called the four-state approach where the model Hamiltonian is built up by a fitting procedure that can be calculated by the supermolecular method coupled with configuration interaction scheme [47]. This method only gives the HOMO to LUMO charge transfer so that usually it is only applicable to the lowest excited state and other charge transfer contributions are neglected. To achieve a more detail study of the first excited state as well as higher excited states in those organic materials, we have applied a full quantum-chemical approach, namely, the Composite-Molecule (CM) model to description the role of CT excitons in low-lying electronic excitations in a number of conjugated systems. Due to the fact that charge-transfer property is often associated with strong interchain interaction, our aim is to discuss the evolution of the charge-transfer interactions associated with the ^1^*B_u_* excited state of a single chain [48,49,50], as a function of both interchain separation and chain length. In fact, it is known that the ^2^*A_g_* state is the lowest excited-state for some short polyene chains [48,49,50]. However, in most common organic conjugated materials, the lowest excited-state is the ^1^*B_u_* state and that is crucial to establish the optical properties in conjugated materials. For the generality, we have investigated the interaction between two ^1^*B_u_* states of those organic dimers.

## 2. Theoretical Models

### 2.1. Composite-molecule (Molecule-in-molecule) method

There is a growing interest for simulation of the chromophore-chromophore interactions in the solid state. An attempt to rationalize the optical properties in the materials (thin films or crystals) is usually based on the calculation of a dimer molecule for simplicity [51,52]. Note that the number of the chromophores usually would not alter the dimer picture in the short chain limit [43], but in the long chain limit the dimer picture would not hold anymore [53]. Nowadays, the molecular exciton model and supermolecular approach have been extensively adopted to predict the changes in optical absorption and luminescence properties of conjugated molecules in condensed phase [54]. In the exciton theory, the excited-state wave functions of the molecules in the solid state are computed only considering the electrostatic interaction. Such an approximation is expected to be valid for weak interchain interactions. In the strong interaction limit, the excited-state wave function would spread out over several molecules and a suitable description of the electronic structure requires the building of delocalized wave functions. In this case, many researchers have adopted the supermolecule approach to accounts for charge-transfer interaction among different chains, which is not the case in the traditional exciton theory. To illustrate this, as given in Equation 1, a delocalized wave function for a dimer can be written as:

(1) $${\text{Ψ}}_{\pm }=c_{1}[\psi (M_{1}*)\psi (M_{2})\pm \psi (M_{1})\psi (M_{2}*)]+c_{2}[\psi ({M_{1}}^{+})\psi ({M_{2}}^{-})\pm \psi ({M_{1}}^{-})\psi ({M_{2}}^{+})]$$

where the terminology for the first term in the field of molecular solids is called Frenkel excitons (local excitons) and for the second term is CT-excitons (charge-transfer excitons). In the exciton theory, only first term is considered whereas both Frenkel excitons and CT-excitons are considered in the supermolecular approach. However, the supermolecular approach does not directly provide the information of relative weights of Frenkel excitons and CT-excitons because of the inadequacy of the basis set construction based on completely delocalized molecular orbitals obtained by the Hartree-Fock calculations. Therefore, we have adopted the composite-molecule (molecule-in-molecule) method which was originally developed by Longuet-Higgins and Murrel for studying excited states of the biphenyl systems based on the fragment molecular orbitals localized on each phenyl group [55]. Warshel and Parson extended this model to investigate the spectroscopic properties of photosynthetic reaction centers [56] and molecular crystals [57]. For organic conjugated materials and cyclophanes, this method is likely to be the most informative if we aim to obtain the dimeric properties from monomeric parameters. Based on the CM theory, the Hamiltonian can be build-up by two non-interacting monomers in which the four diagonal blocks stand for localized Frenkel exciton, CT-exciton, hole-transfer, and electron-transfer subspaces, and the off-diagonal blocks represent the interaction among them. The localized Frenkel-exciton subspace will be first partially diagonalized by an intermediate transformation [56], such that the dimer wavefunctions can be written in terms of the superposition of Frenkel- and CT-exciton instead of the local configuration-state functions.

The molecular orbitals (*φ*) of the individual molecules are written as linear combinations of atomic orbitals in Equation 2. The expansion coefficients (*C*) of monomers I and II in the dimer are obtained by solving the corresponding Hartree-Fock equations of each isolated fragments [47]. The basis function *χ_p_* and *χ_q_* are taken to be the p_z_ atomic orbital on the atom *p* and *q*, respectively. The interaction between the two chromophores in a dimer is introduced at the level of configuration interaction by constructing a CI matrix (**A**) with the matrix elements given by

(2) $$\varphi_{n}^{I}=\sum_{p}^{I}C_{n,p}\chi_{p}^{I}\;\text{and}\;\varphi_{m}^{II}=\sum_{q}^{II}C_{m,q}\chi_{q}^{II}$$

(3) $${\text{A}}_{N,M}=\delta_{na,ma}{\text{F}}_{nr,mr}-\delta_{nr,mr}{\text{F}}_{na,ma}-\langle m_{a}n_{r}|n_{a}m_{r}\rangle +2\langle m_{a}n_{r}|m_{r}n_{a}\rangle$$

where N and M stand for singly excited configuration functions of chromophores I and II, respectively. *δ* is the Kronecker delta function and the indices, a and r, indicate the occupied and unoccupied molecular orbitals, respectively. The matrix elements of **F** matrix are given by Equation 4; while the two-electron integrals can be evaluated by using Equation 5 within the zero-differential overlap (ZDO) approximation. The *r*_12_ is the distance between electron 1 and 2 and γ is the two-center electron repulsion integral.

(4) $${\text{F}}_{nj,mk}=\sum_{p}^{I}\sum_{q}^{II}C_{nj,p}C_{mk,q}\langle \chi_{p}|H_{p,q}^{core}|\chi_{q}\rangle =\sum_{p}^{I}\sum_{q}^{II}C_{nj,p}C_{mk,q}\beta_{p,q}$$

(5) $$\begin{array}{l} \langle m_{a}n_{r}|n_{a}m_{r}\rangle =\iint \varphi_{ma}^{II}(1)\varphi_{nr}^{I}(2)(1/r_{12})\varphi_{na}^{I}(1)\varphi_{mr}^{II}(2)d\tau_{1}d\tau_{2}\\ \approx \sum_{p}^{I}\sum_{q}^{II}C_{ma,q}C_{nr,p}C_{na,p}C_{mr,q}\gamma_{p,q} \end{array}$$

Subsequently, we apply the intermediate transformations on **A** matrix such that the local exciton subspaces are diagonalized in the local exciton wavefunctions,

(6) $$\Psi_{i}^{I}=\sum_{N}^{I}{\xi_{i,N}}^{1}\psi_{N}^{I\to I}\;\text{and}\;\Psi_{k}^{II}=\sum_{M}^{II}{\xi_{k,M}}^{1}\psi_{M}^{II\to II}$$

where $\psi_{N}^{I\to I}$ and $\psi_{N}^{II\to II}$ are the local excited configurations involving removing one electron from an occupied to an unoccupied orbitals of monomers I and II, respectively. The configuration interaction (CI) expansion coefficients (*ξ*) are obtained by solving the CI matrix [47]. The exciton-interaction matrix can be described by Columbic, exchange and penetration terms [58,59] where the exchange term is zero when ZDO approximation is applied. Penetration are short-range processes, which scales as the overlap between the two-chromophore orbitals (*i.e.*, decay exponentially with distance) The conjugated chromophores with interchain interaction or intrachain chain-end interaction have been examined that Columbic interaction is good enough for describing the energy transfer process [59]. Therefore, here, we consider the most important Columbic term as given in Equation 7. The remaining submatrices in the CM model can be estimated [47].

(7) $${\;}^{\text{U}}{\text{A}}_{i,k}^{I,II}=\langle {\text{Ψ}}_{i}^{I}|\text{H}|{\text{Ψ}}_{k}^{II}\rangle =2\sum_{N}^{I}\sum_{M}^{II}\sum_{p}^{I}\sum_{q}^{II}\xi_{i,N}\xi_{k,M}C_{ma,p}C_{mr,p}C_{na,q}C_{nr,q}\gamma_{p,q}$$

**Scheme 1:**
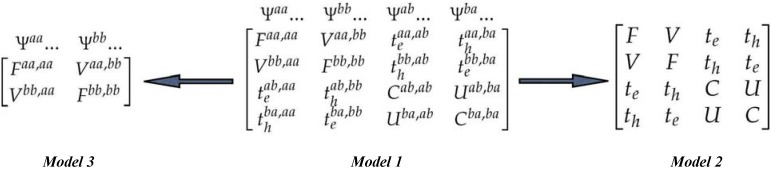
CM Hamiltonian matrix (model 1), truncated four-orbital CM model (model 2) and truncated CM model when CT transitions are omitted from the calculation (model 3) [46]. Reproduced with permission from American Chemical Society.

Based on the CM Hamiltonian, the four diagonal blocks stand for Frenkel exciton (**F**), CT-exciton (**C**), electron-transfer (**t_e_**), and hole-transfer (**t_h_**) subspaces, and the off-diagonal blocks (**V** and **U**) represent the interaction among them (model 1 in Scheme 1). Thus, the wavefunctions of the dimers are expressed in terms of a superposition of a local excitons and charge resonance configurations

(8) $${\text{Ψ}}_{\pm }^{CM}=\frac{1}{\sqrt{2}}\sum_{i}c_{\pm ,i}^{ex}[\psi_{i}(M_{1}*)\psi_{0}(M_{2})\pm \psi_{i}(M_{1})\psi_{0}(M_{2}*)]+\frac{1}{\sqrt{2}}\sum_{a,r}c_{\pm ,ar}^{ct}[\psi_{a}({M_{1}}^{+})\psi_{r}({M_{2}}^{-})\pm \psi_{a}({M_{1}}^{-})\psi_{r}({M_{2}}^{+})]$$

where *ψ_i_*(*M*^∗^) is the *i*-th singlet state of monomer *ψ*_o_(*M*) and *ψ_a_*(*M*^+^)*ψ_r_*(*M*^-^) (*ψ_a_*(*M*^−^)*ψ_r_*(*M*^+^)) corresponds to a charge-transfer configuration in which an electron moves from local molecular orbital *ϕ_a_* of the monomer in a dimer to unoccupied orbital *ϕ_r_* of the other one. The percentage of exciton resonance component and charge resonance component can be calculated

(9) $$\omega_{LE}={\sum_{i}\left|c_{i}^{ex}\right|}^{2},\;\omega_{CT}={\sum_{a,r}\left|c_{ar}^{ct}\right|}^{2}$$

for which *w_LE_* + *w_CT_* = 100%. Therefore, the relative weights *w_LE_* and *w_CT_* stand for a useful measure of interchain interaction for the two moieties in a dimer, which are hard to obtain from a supermolecular approach.

To abstract the importance of the charge-transfer contribution to the low-lying two excitation energies, two truncated models are compared with the CM Hamiltonian (model 1). In model 1, the CM Hamiltonian matrix of the dimer is constructed by considering all intrachain and interchain transitions within the single configuration-interaction (SCI) scheme [60]. In a next step, we have truncated this Hamiltonian to four essential frontier orbitals (model 2); that is, HOMO and LUMO orbitals on one chain and another two orbitals on the other chain. Therefore, in this model, only HOMO to LUMO charge-transfer is considered. The matrix of model 3 can be constructed by the model 1 with all CT transitions are omitted from the calculation. Based on these three models, we can elucidate the importance of the lowest CT and other CT transitions contributions to the lowest two excited states.

### 2.2. Truncated composite-molecule (Molecule-in-molecule) method

**Scheme 2:**
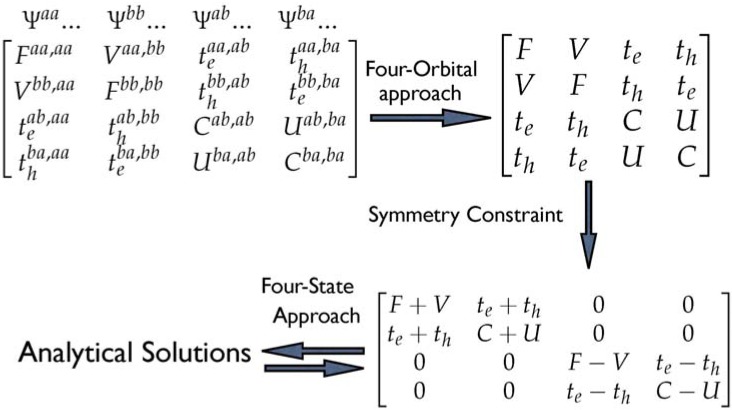
The truncated MIM model with symmetry constraint [47]. Reproduced with permission from American Chemical Society.

Here, we introduce a simplified four-state method based on the truncated MIM Hamiltonian as shown in Scheme 2, which allows us to obtain the relative contributions of the Frenkel (local) and charge-transfer (CT) excitons in the supermolecular calculations. The truncated MIM Hamiltonian of a molecular dimer at SCI level is constructed by a minimal CI matrix, which includes only the most important transitions (two local and two CT excitons) among four essential frontier orbitals. The diagonal matrix elements F and C are Frenkel and a CT exciton, respectively. There are two kinds of the off-diagonal matrix elements. First, the exciton-exciton interaction among two localized Frenkel (CT) exciton, V and U, is responsible for the exciton delocalization. Second, the matrix elements, t_h_ and t_e_, are the hole and electron transfer coupling, respectively. The truncated MIM Hamiltonian can be block-diagonalized with the C_2_ symmetry of the molecule, and then the analytical solutions can be obtained by solving the resulting quadratic equations. Combining the energies of excited states calculated by quantum chemical methods (INDO/S, TD-DFT, *etc.*) with the analytical solutions, this four-state approach can be used to estimate the matrix element F, C, V, and U in the effective MIM Hamiltonian. The four excited states, characterized by the symmetries of the configuration description, only two sets of the configurations are expected, the first set includes the HOMO ➔ LUMO and HOMO-1 ➔ LUMO + 1 transitions (E_f1_ and E_f2_ in the order of increasing energy), the other set contains the HOMO ➔ LUMO + 1 and HOMO - 1 ➔ LUMO transitions. (E_a1_ and E_a2_) The estimation of the electronic coupling can be obtained by a number of computational techniques [63,64,65,66]. One widespread approach is to estimate the transfer integral for holes (electrons) as half the splitting of the HOMO (LUMO) levels [67]. The applicability of this method was in good agreement with many other *ab-initio* calculations [68,69,70]. Therefore, this method can be used to estimate the hole (electron) transfer matrix elements for a dimer. To evaluate the coupling reasonably, we have checked the electron distribution in the essential four orbitals by the symmetric and antisymmetric combinations of the two single chromophore units.

It is noteworthy that this method still holds in cyclophane systems where hyperconjugation between chromophores and tethers is relatively weak, which we will discuss in detail in Section 4. Within this methodology, the matrix elements of the CT-exciton (C) and its coupling (U) in this four-state model can be found as

(10) C=(1/2)(Γ+Ω)

(11) U=(1/2)(Γ−Ω)

where Γ and Ω are

(12) $$\text{Γ}=(1/2)[(E_{a1}+E_{a2})+\sqrt{{\left(E_{a1}-E_{a2}\right)}^{2}-4{\left(t_{e}+t_{h}\right)}^{2}}]$$

(13) $$\text{Ω}=(1/2)[(E_{f1}+E_{f2})+\sqrt{{\left(E_{f1}-E_{f2}\right)}^{2}-4{\left(t_{e}-t_{h}\right)}^{2}}]$$

Furthermore, the Frenkel exciton (F) and exciton coupling (V) can also be determined as

(14) $$F=(1/2)(E_{a1}+E_{a2}+E_{f1}+E_{f2})-C$$

(15) $$V=(1/2)(E_{a1}+E_{a2}-E_{f1}-E_{f2})-U$$

Combining transfer integrals, t_e_ and t_h_, with the energies, E_a1_, E_a2_, E_f1_, and E_f2_, of low-lying excited states obtained from the supermolecular SCI calculation, Eqs. 10-15 can be used to determine matrix elements F, U, C and V. With all these matrix elements estimated, we can calculate the CT contributions by this simplified model.

### 2.3. Computational methodology

In Section 3, all the ground state structure of all monomers were computed using the semiempirical Hartree-Fock Austin Model 1 (AM1) method [71]. The excited-state wavefunctions of the monomer were obtained by the single configuration-interaction (SCI) scheme [60] with all occupied and unoccupied π-levels included within the semiempirical Pariser-Parr-Pople (PPP) Hamiltonian [56,57,72] which has been used extensively in the studies of conjugated molecules, and has given reliable results for spectroscopic and other linear or nonlinear optical properties when compared with experimental results and computationally highly extensive *ab-initio* calculations [73,74,75]. The reason why we chose this simple p-orbital based PPP model is that it is believed that the electronic and optical properties of conjugated molecules lie in the p orbitals of the backbone atoms, which overlap to form delocalized p molecule orbitals [76]. The parameters that we used for our PPP calculations are collected from various sources in the literature in a fairly consistent fashion.[47,77,78] Besides, our PPP calculation has been shown to be well reproduced the excitation energies as well as excited-state properties compared with the all-valence INDO/S method [47,79], which has been demonstrated to provide consistent results of excitation energies and molecular orbitals energies in comparison with the UV/Vis spectrum and ultraviolet photoelectron spectroscopy (UPS) experimental observations of conjugated molecules, respectively [80,81].

In Section 4, the ground state structure of cyclophanes were computed using the semiempirical Hartree-Fock Austin Model 1 (AM1) method [71] and the optimized geometries obtained were usually in good agreement with the X-ray structures [82,83,84]. In our study, we optimized the model cyclophanes by using both the semiempirical AM1 method and the density functional theory (DFT) method to prevent the potential artifact of the computational methods adopted [85]. In the density functional theory (DFT) method, we employed the B3LYP functional, where Becke’s three-parameter hybrid exchange functional is combined with the Lee-Yang-Parr correlation functional [86,87,88]. All DFT calculations were carried out with the 6-31G** split valence plus polarization basis set [89,90,91,92,93]. According to other reports, this method may provide reasonable cyclophane structures compared with X-ray data [94] and other *ab-initio* results [95]. The excitation energies and electronic structures were calculated by the single configuration-interaction (SCI) [60] scheme with all occupied and unoccupied π-levels included within the semiempirical intermediate neglect of differential overlap (INDO) model [79], as parameterized by Zerner and co-workers [80].

## 3. Charge-Transfer Interactions in Organic Materials

### 3.1. Charge-transfer interactions in polyenes

**Figure 1:**
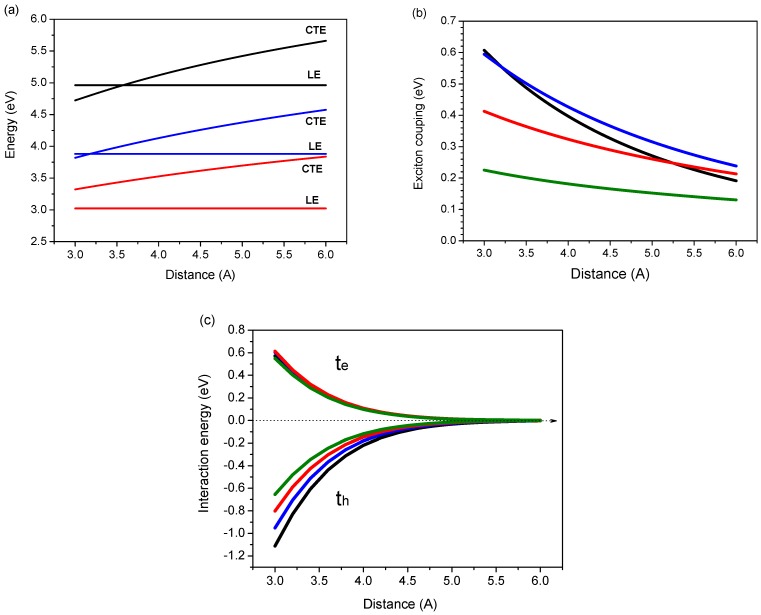
Distance dependence of the calculated **(a)** local exciton (LE) and charge-transfer exciton (CTE) energies **(b)** exciton coupling (2V) and **(c)** interaction energies of the electron transfer (t_e_) and hole transfer (t_h_) of various polyene dimers, with N = 6 (black), 10 (blue), 20 (red), and 40 (green) based on the four-orbital CM model (model 2 in Scheme 1) [46]. (Note that although we provide a detail distance dependence data, the van der Waals radius of carbon is 1.7 Å). Reproduced with permission from American Chemical Society.

Our goal in this section is to illustrate how interchain distance and chain size alter the interchain interaction. This is exemplified below via a detailed comparison of the electronic structure of polyene dimers. Specifically, we investigate the evolution of the CT excitons as well as the interaction between local excitons and CT excitons in cofacial polyene dimers with chain size (up to 40 carbon atoms) and interchain separation (3-6 Å) by model 2. It is known that the CT excitons lie well above the local excitons when the interchain distance is large compared to the molecule size. However, for small interchain separations, CT excitons can be expected to be close or lower than that of the local excitons. The energy of the CT excitons as a function of interchain distance in cofacial polyene dimers is shown in Figure 1a. Note that the typical interchain separation of conjugated molecules in the solid state is known to be around 4 Å. It is clear that the mixing of CT exciton is sensitive to the interchain distance and the CT excitons could be lower than the local excitons for small interchain separations. For instance, when the interchain distance of the 6-site polyene dimer is shorter than 3.6 Å, the energy of CT excitons is lower than that of the local exciton. It is noteworthy that with increasing molecule size, the CT excitons lie above the local excitons.

The exciton couplings of polyene dimers have been estimated by a number of methods such as the point-dipole approximation, the extended dipole approximation, and the supermolecular calculations at the semiempirical level [54,97,98]. Recently, it has been shown that for conjugated systems, this semiempirical method is adequate to describe the exciton coupling when compared with the *ab initio* calculations [52]. The calculated exciton coupling energy (2V) as a function of the interchain separations of selected polyene dimers is shown in Figure 1b. The magnitude of the exciton coupling increases with decreasing interchain distance. For example, the polyene dimer with N = 6, those exciton couplings are d = 4 Å, 0.40 eV to d = 6 Å, 0.19 eV which are consistent with the previous ab inito calculations (d = 4 Å, 0.51 eV to d = 6 Å, 0.30 eV). More importantly, for a range of interchain separations, it reaches a relative maximum for 10-site polyene dimer in comparison with those of 6-, 20-, and 40-site polyene dimers. These calculated magnitude and trend of the exciton coupling are consistent with the results reported in the literature [54,97,98].

From Scheme 2, the interaction between the local exciton and the charge-transfer exciton can be characterized by the electron- and hole-transfer integrals. In the strong interaction limit, these charge transfer interactions are expected to be large because they depend on the spatial overlap between the molecular orbital wave functions [99]. The evolution of electron- and hole-transfer integrals as a function of interchain distance for selected polyene dimers are shown in Figure 1c. These results based on directly calculating the matrix elements of transfer intergrals are consistent with the energy-splitting estimates provided by earlier INDO calculations [100]. For instance, the polyene dimer with d = 4 Å, those hole transfer couplings are 0.18 eV for N = 6 and 0.15 eV for N = 10 which are consistent with the INDO calculations (0.16 eV for N = 6 and 0.14 eV for N = 10). It is important to note that even in a dimer constructed by two identical monomers, the transfer integrals estimated by the energy-splitting method can be affected by the difference of the site energies which is induced by polarization effect [101,102]. In the case of a cofacial dimer, the polarization effects can be ignored because of symmetry. It is clear from Figure 1c that the hole transfer interaction in 6-site polyene dimer is larger than that calculated for the electron. This feature is still holds when the chain size increases but the difference in interaction energies converge towards the same value.

**Scheme 3:**
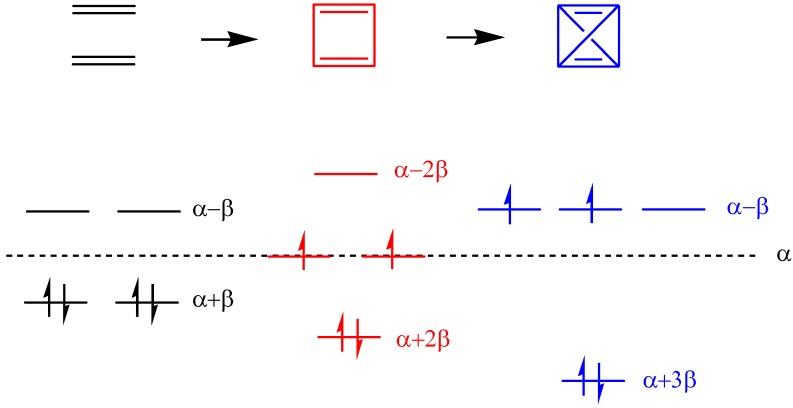
Energy diagram of the interactions between two ethylenes based on simple Hückel model [46]. Reproduced with permission from American Chemical Society.

Larger hole transfer interaction than that of electron as well as the chain length dependence usually can be rationalized by a simple way which is related to the number of nodal planes in the MOs [100]. However, we found that the larger hole-transfer interaction might be attributed to not only wave function phase (nodal plane) but also interchain next-nearest neighbor interaction. To illustrate this, we have adopted a very simple way to rationalize this result by considering the energy-splitting method and Hückel model (Scheme 3). Without the interchain interaction between two ethylene monomers, the electron- and hole-transfer integrals are zero. Further, if the interchain nearest-neighbor interactions in the ethylene dimer (cyclobutadiene) are considered, both the electron- and hole-transfer integrals are equal to β. Subsequently, considering both the interchain nearest and next nearest neighbor interactions, the electron-transfer integral is zero and the hole-transfer integral is 2β. Note that although the interchain next-nearest neighbor interaction should be smaller than the nearest neighbor interaction which is due to smaller overlap integral, the general picture is depicted from this approach.

To investigate this issue, the magnitude of the matrix elements of electron- and hole-transfer integrals in a cofacial ethylene dimer are equal to Equations 4 and 5, respectively, which are obtained based on perturbation theory [99].

(16) $$t_{e}=\langle \varphi_{LUMO}^{1}|h|\varphi_{LUMO}^{2}\rangle =\sum_{\mu }\sum_{\nu }C_{LUMO,\mu }C_{LUMO,\nu }\langle \chi_{\mu }^{1}|h|\chi_{\nu }^{2}\rangle$$

(17) $$t_{h}=\langle \varphi_{HOMO}^{1}|h|\varphi_{HOMO}^{2}\rangle =\sum_{\mu }\sum_{\nu }C_{HOMO,\mu }C_{HOMO,\nu }\langle \chi_{\mu }^{1}|h|\chi_{\nu }^{2}\rangle$$

In Equations 16 and 17, the *φ*_LUMO_ and *φ*_HOMO_ denote the LUMO and HOMO molecular orbitals for individual chains, respectively, and χ_μ_is the atomic orbital centered on atom μ. If the interchain nearest neighbor interaction is β and interchain next-nearest neighbor interaction is β’, the corresponding electron and hole transfer integrals for the ethylene dimer are 2C^2^(β-β’) and 2C^2^(β+β’), respectively. Therefore, the difference between electron and hole transfer interactions is attributed to the positive and negative sign and β’. The sign results from the phase of wave function and β’ is the interchain next-nearest neighbor interaction. Thus, the interchain next-nearest neighbor interaction is a key factor to result in different transfer interaction energies. Accordingly, we emphasize that the key factors for the difference in the hole- and electron coupling should be the cooperative effect induced by the wave function phase (nodal plane) and the interchain next-nearest neighbor interaction.

**Scheme 4:**
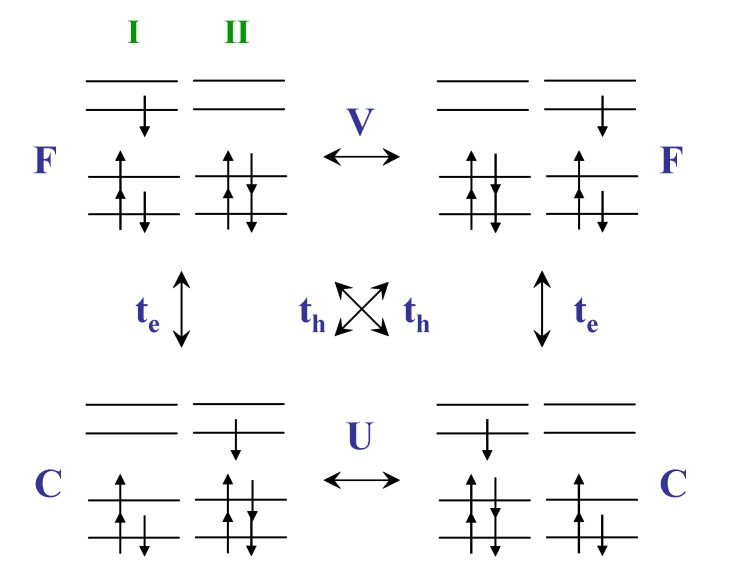
Simplified configuration interactions in the excited state for a dimer based on four-orbital model. The definition of all symbols is described in Section 2.

**Figure 2:**
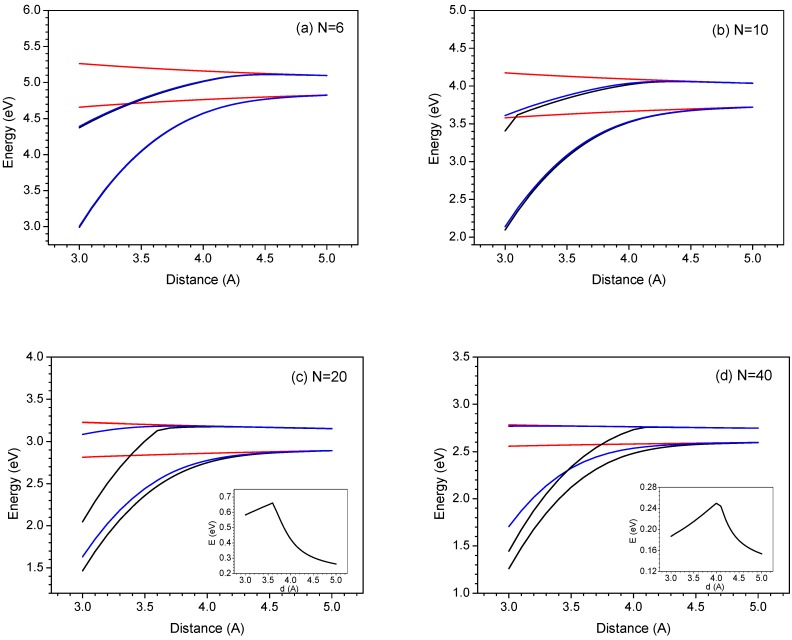
Distance dependence of the two low-lying excited states of various polyene dimers, with N = 6 **(a)**, 10 **(b)**, 20 **(c)**, 40 **(d)** calculated by model 1 (black), model 2 (blue) and model 3 (red line). The insets show the evolution of the energy gap of the two transitions as a function of the intermolecular distance (calculated by model 1). For clarity the data calculated by model 3, we specify the exact values at 3.0 and 5.0 Å for N = 6 (S_1_:4.657 eV, 4.827 eV; S_2_:5.264 eV, 5.098 eV), 10 (S_1_:3.580 eV, 3.722 eV; S_2_:4.174 eV, 4.037 eV), 20 (S_1_:2.814 eV, 2.893 eV; S_2_:3.227 eV, 3.153 eV), 40 (S_1_:2.558 eV, 2.596 eV; S_2_:2.784 eV, 2.748 eV) [46]. Reproduced with permission from American Chemical Society.

The excited state wave function can be written by linear combination of the four configurations as shown in Equation 1 and Scheme 4. Note that the delocalized dimer wave function is consisted of two Frenkel and two charge transfer configurations. It is important that the interactions between those configurations are distance dependence as shown in Figure 1b and 1c. Therefore, when the distance between two chromophore decreases, one can expect that the excitation energy would be perturbed by the charge transfer contributions via electron- and hole-transfer interactions. Now, we first address the effect of the charge-transfer interactions on the two low-lying singlet transitions. In Figure 2, we display the evolution of the two excitation energies of the N-site polyene dimers (N = 6, 10, 20 and 40) as a function of interchain distances. To illustrate the importance of the HOMO to LUMO charge-transfer as well as other charge-transfer contributions to the two singlet excitation energies, we have compared the calculation results based on models 1-3 which have been described in the methodology section. Note that excitations computed by model 3 can be viewed as references where only electrostatic interactions between two chains are included in the calculations so that the larger CT contributions, the larger the deviations between the results calculated by model 1 (model 2) and model 3. In model 3, as shown in Figure 2, the first (second) transition energies of all the polyene dimers decreases (increases) monotonically with decreasing the interchain distances. These results can be rationalized by a simple exciton interaction picture [100]. Now, we consider model 1, where both the two excitation energies matches well with the transition energies calculated by model 3 when interchain distance larger than 4.5 Å but a large deviation was found when the two polyene chains are close in space (d < 4.5 Å). Recall the results in Figure 1: the energy of the charge-transfer exciton is close to that of local exciton when the interchain distance is lowered from 4.5 Å to 3 Å. Moreover, that electron and hole couplings among local exciton and charge-transfer exciton are also amplified in this strong interaction region (see in Figure 1c). Based on these results, the charge-transfer transitions would reasonably affect the excitation energies when the interchain separations are smaller than 4.5 Å. In the strong interaction region (d < 4.5 Å), both transition energies of the 6- and 10-site polyene dimers decrease with decreasing the interchain distances with respect to the transition energies calculated by model 3. For 20- and 40-site polyene dimers, the evolution of the first transition energies is similar to those of shorter polyene dimers. However, the deviation of the second transition of the 20- and 40-site polyene dimers appears around 3.6 Å and 4.0 Å, respectively. When the interchain distances are smaller than that, it is interesting to note there is a sharp decrease of the second transition energy which is even more sensitive than the first transitions. The energy gaps between the two excited states show a peak behavior with an increase followed by a drop for 20- and 40-site polyene dimers; see the inset of Figure 2. These results indicate that there is a significant discrepancy in the two excitation energies with respect to the excitation energies calculated by model 3. Therefore, the charge-transfer interaction should play a critical role for the distance dependence of the two excitations in those polyene dimers.

In comparing to the transition energies calculated by model 1 and model 2, it is clear that only HOMO to LUMO charge-transfer transition contributes to the first excited state for 6-site polyene dimers. With increasing size of the polyene chains, the HOMO to LUMO configuration still holds major contribution to the lowest excitation energy whereas some discrepancy was found that can be attributed to the contribution from other higher energy charge transfer configurations. On the other hand, the influence of the HOMO to LUMO charge transfer transition on the second excited-state is quite sensitive to the polyene chain lengths. For short chains such as 6-site polyene, the second excitation energy is mainly due to HOMO to LUMO charge transfer. However, the second excited-states of 40-site polyene dimer calculated by model 2 and model 3 are superimposed, which indicate that the HOMO to LUMO charge-transfer only has a negligible contribution to the second excitation energy. Thus, when the interchain separation is smaller than 4 Å, there is a significant charge-transfer contribution to lower the second excitation energy which is attributed to charge-transfer transitions with higher energy than that of HOMO to LUMO.

According to these results, we conclude that in the strong interaction limit, the evolution of the two transition energies for 6- and 10-site polyene dimers is attributed to the HOMO to LUMO charge-transfer interactions. For the 20- and 40-site polyene dimers, in the region of increasing the energy gap between two transitions, these results can be explained by (a) lowering the first excitation energies and (b) the HOMO to LUMO charge-transfer transition being the major and partial contributions that affect the transition energies. On the other hand, in the region of decreasing energy gap between two excited states, we conclude that this behavior mainly arises from lowering the second excitation energy where the major charge-transfer contribution is not from HOMO to LUMO charge-transfer transition. When comparing the second excitation energies calculated by model 1 and model 3 as shown in Figure 2, it is interesting to note that there is a non-negligible charge-transfer contribution for 10- and 40-site polyene dimers with interchain separation by around 3.6-4.0 Å, but it is not the case for 20-site polyene dimer which indicates that there is a minimum charge-transfer contribution to the second excitation energy with appropriate chain size and interchain separation. Based on the calculation results described above, we conclude that in the weak interaction region (d > 4.5 Å), the truncated model 3 is applicable because the contribution of the CT exciton is negligible small. On the contrary, in the strong interaction region (d < 4.5 Å), the truncated model 2 is applicable only for smaller polyene dimers, because many higher lying configurations would contribute to the second excited state when larger chromophores are considered. Thus, model 1 is necessary for calculating the second transition energy of a given polyene dimer with longer chain length. Moreover, in the strong interaction region, the simple four orbital model (model 2) is useful to describe first excitation energy of N-site (N < 20) polyene dimers. With increasing the chain length, for example 40-site polyene dimers, there are some contributions from other charge transfer transitions that can obviously lower the transition energy. For the second transition, the number of essential charge transfer configuration increases with increasing chain size as the spacing between higher excited states decreases. In this case, the four orbital model only can be applicable to shorter N-site (N < 10) polyene dimers based on our calculations.

**Figure 3:**
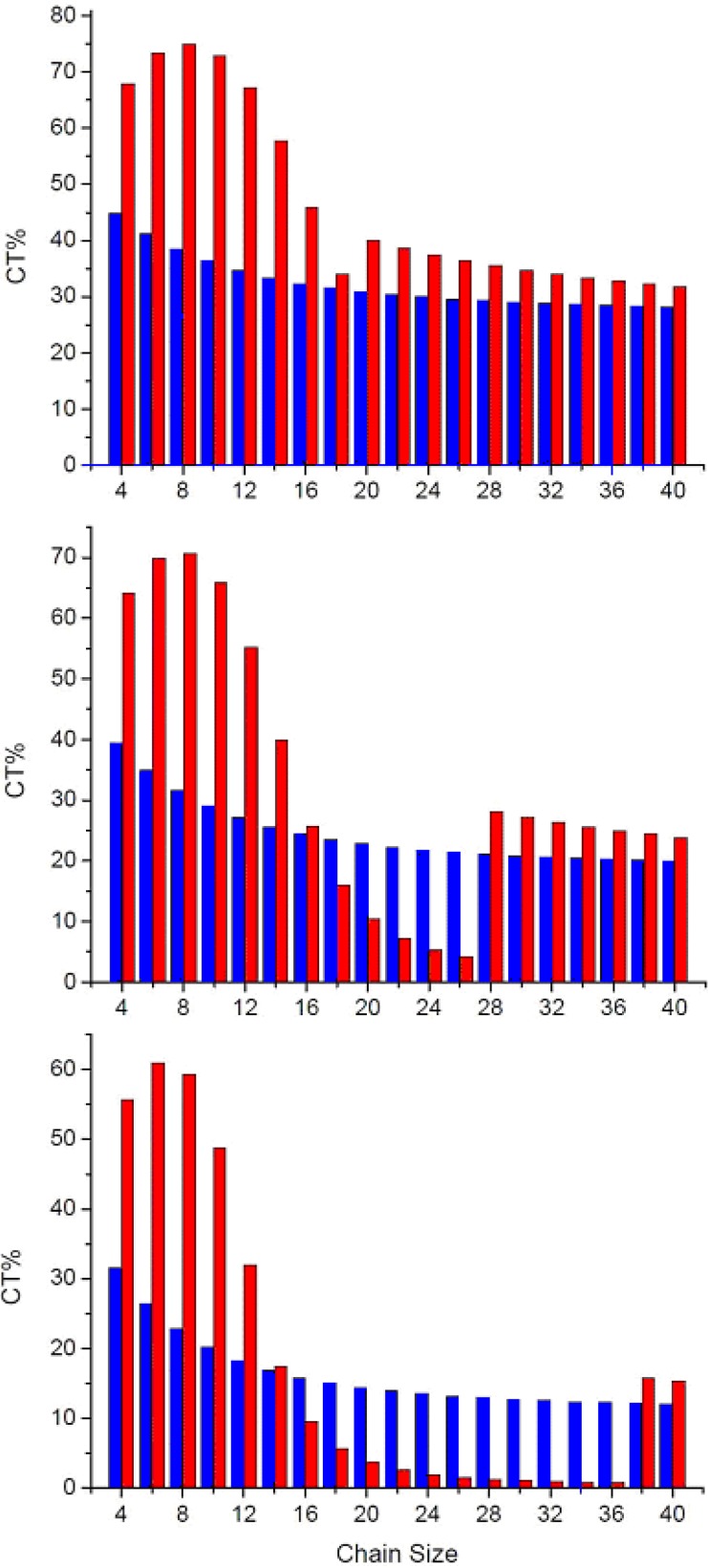
Dependence of the CT wave function (percentage) of lowest excited-state (blue) and second excited-state (red) as a function of chain size, with interchain distance d = 3.6 Å (upper), 3.8 Å (middle), 4.0 Å (lower), calculated by the CM model [46]. Reproduced with permission from American Chemical Society.

Low-lying transitions of a dimer are closely related to optical properties of the organic materials in the solid state [100]. For example, the CT contribution to the first excited state is usually related to the efficiency of the organic light-emitting diode (OLED) materials because the CT exciton is detrimental to the quantum yield of the material and its contribution to the second excited state is possibly related to the efficiency of the solar cell materials (the CT contribution is believed to increase the efficiency of the material) for the reason that when considering a face-to-face packing dimer with appropriate interchain distance, the second transition is one-photon allowed [100]. Therefore, the comparison of the charge-transfer contributions of the two excited states may provide an insight into which application is possible from the perspective of a given chemical dimer structure since the number of chromophores usually would not alter the dimer picture in the short chain limit [43]. To understand this in detail, in Figure 3, we have investigated the dependence of the charge-transfer wave function (in percentage) in the two excited states as a function of chain size calculated by the model 1. Table 1 shows that the magnitude of the CT% to the first excitation is monotonically decreased with the chain size as well as the interchain separation. The CT% contribution to the second excited state first increases with chain length, reaches a maximum, and then decreases for longer conjugated segments to reach a minimum.

**Figure 4:**
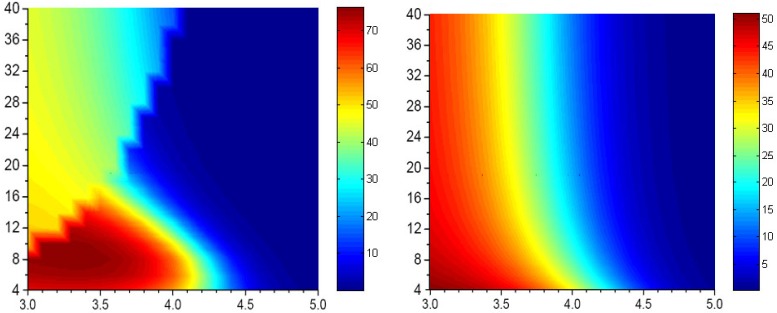
Contour polts of the CT exciton (in percentage) as a function of interchain distance (x axis) and chain size (y axis) of the first (right) and second (left) excited states in polyene dimers calculated using model 1 [46]. Reproduced with permission from American Chemical Society.

There is a window for which the contribution of the CT% to second excited is smaller than that of first excited state when the interchain distance is 3.8 and 4.0 Å. More importantly, with further chain size increases after the CT% reaches the minimum, the CT% contribution to the second excitation was suddenly increased and then decreased monotonically thereafter. For instance, the jump points appear at 28- and 38-site polyene dimers with interchain distance of 3.8 and 4.0 Å, respectively. These results indicate that besides the HOMO to LUMO charge transfer, there is a new charge transfer contribution to the second excited state and this contribution is sensitive to the chain size and interchain distance. The contribution of the new charge-transfer transitions to the second excited state can be even larger than HOMO to LUMO contribution to the first excited state. Based on the calculation results, we can envision that for a given organic material with larger conjugated length, the charge-transfer contribution to the second transition in a cofacial packing might be due mainly to this contribution mainly. For example, the contribution of charge transfer interaction in the higher-lying excited state of porphyrin molecules has been studied recently [103,104,105,106,107]. To get a deeper insight into the origin of the CT contributions to the lowest two excited states in the polyene dimer, we have examined the configuration-state function for the polyene dimer with interchain separation by 3.8 Å in Table 1. The major CT contribution to the first excited state is from HOMO to LUMO and then monotonic decreases with chain size. For the second transition, the HOMO to LUMO charge transfer configuration holds the major CT contribution for short chain polyene dimer. With increasing the chain size, this CT contribution decreases but the CT contribution decreases and the CT% from two CT configurations suddenly increase (these are HOMO-1 to LUMO and HOMO to LUMO + 1 charge-transfer configuration-state functions). These transitions are similar to the porphyrins’ excitations because of symmetry [43,44,56,108,109,110,111]. It is useful to have a closer look at the CT% distribution in the lowest two excited states. In Figure 4, we display the contour plots of the CT% as a function of chain size and interchain separation. The calculated chain-length and interchain separation dependence of the CT% for the first excited state showing higher CT% appears when smaller chain length and interchain separation are considered. The CT% distribution of the first excited state is almost lower than 50%. For the second excited state, it is significant that there is a region where the CT% higher than 50%. The calculated CT% shows a maximum around 8-site polyene dimer with interchain distance 3.3 Å and the CT% reaches 75%. Interestingly, there is a region where the chain-length dependence of CT% exhibits a minimum with interchain separation around 3.6-4.0 Å.

**Table 1:** Calculated electronic transitions and state functions for cofacial polyene dimers separated by 3.8 Å [46]. Reproduced with permission from American Chemical Society.

N-site	Ε *^a^*	Ε *^b^*	CT% *^a^*	Major CT-state function *^a^*
6	4.40 (E_1_)	4.40 (E_1_)	35.0	0.42 H_M1(M2)_ → L_M2(M1)_ (100%)*^c^*
	4.91 (E_2_)	4.91 (E_2_)	69.9	0.59 H_ M1(M2)_ → L _M2(M1)_ (99.6%)
10	3.38 (E_1_)	3.38 (E_1_)	29.1	0.38 H _M1(M2)_ → L _M2(M1)_ (99.2%)
	3.95 (E_2_)	3.96 (E_2_)	65.9	0.57 H _M1(M2)_ → L _M2(M1)_ (98.6%)
20	2.64 (E_1_)	2.64 (E_1_)	22.9	0.33 H _M1(M2)_ → L _M2(M1)_ (95.2%)
	3.17 (E_2_)	3.17 (E_2_)	10.5	0.22 H _M1(M2)_ → L _M2(M1)_ (92.2%)
30	2.46 (E_1_)	2.46 (E_1_)	20.9	0.29 H _M1(M2)_ → L _M2(M1)_ (80.4%)
	2.84 (E_2_)	2.84 (E_2_)	27.2	0.24 H-1 _M1(M2)_ → L _M2(M1)_, 0.25 H _M1(M2)_ → L+1 _M2(M1)_ (88.3%)
40	2.39 (E_1_)	2.39 (E_1_)	20.0	0.27 H _M1(M2)_ → L _M2(M1)_ (72.9%)
	2.62 (E_2_)	2.62 (E_2_)	23.9	0.22 H-1 _M1(M2)_ → L _M2(M1)_, 0.22 H _M1(M2)_ → L+1 _M2(M1)_ (81.0%)

*^a^* Calculated by CM method (model 1) and Energy in eV; *^b^* Calculated by supermolecular model.[47,54]; *^c^* Major CT Contribution to the overall CT wavefunction (in percentage).

**Figure 5:**
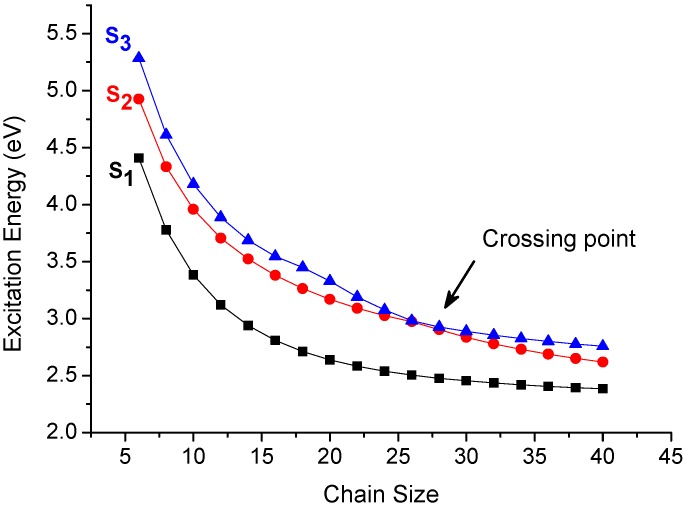
Chain size dependence of the three low-lying excited states of polyene dimers with interchain distance of 3.8 Å (calculated by model 1).

In order to avoid the artifact of the sudden increase of the CT% in Figure 3, we have performed the supermolecular calculations on polyene dimers with various chain sizes and the data is collected in Table 1. In comparison of the excitation energies calculated by CM method (model 1) with the supermolecular approach, these results suggest these excitations calculated by CM model are perfectly consistent with the supermolecular calculations, which means state functions of these transitions should not result from an artifact. To understand the data reasonably, we also analyze the lowest three excited states as a function of chain size (Figure 5). It is interesting to note that there is a crossing point for 28-site polyene dimer. This result reveals that it is reasonable to have a sudden change of the CT% of the second excitation which is due to exchange of two electronic states and that is consistent with the sudden increase of CT% of second excitation for 28-site polyene dimer in the middle of Figure 3.

### 3.2. Charge-transfer interactions in OPV, OT and OP

**Figure 6:**
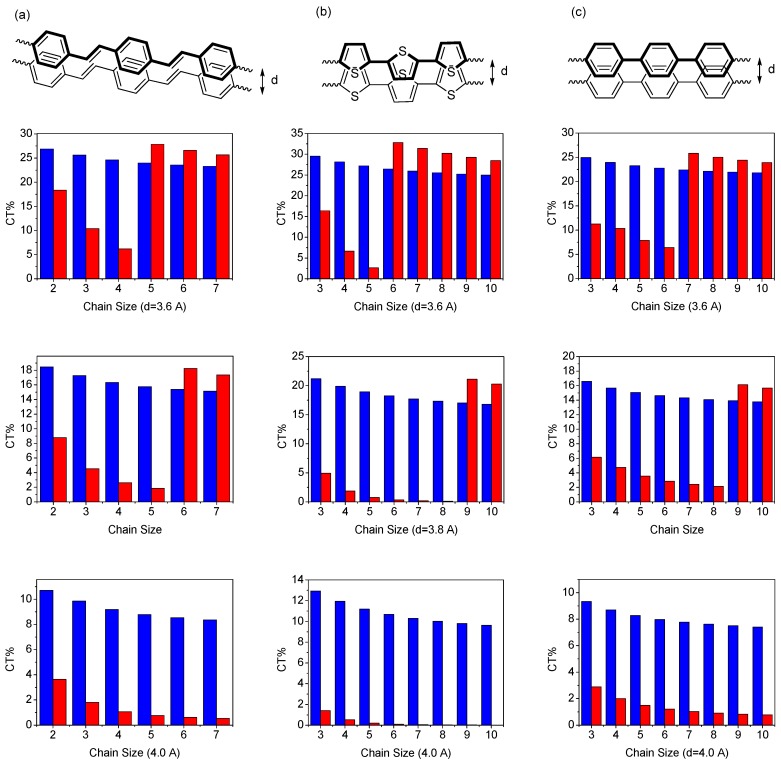
Dependence of the CT wave function (percentage) of lowest excited-state (blue) and second excited-state (red) for **(a)** PV_n_ **(b)** T_n_ and **(c)** P_n_ systems as a function of chain size, with interchain distance d = 3.6 Å (upper), 3.8 Å (middle), 4.0 Å (lower), calculated by the CM model.

To check the general applicability of the conclusions above to more practical organic materials, in the following we apply the CM method to identify the CT contributions to the low-lying singlet excited states in a number of well-known organic materials, including oligophenylenevinylenes (OPV_n_), oligothiophenes (OT_n_), and oligophenylenes (OP_n_). Based on our previous study on polyene system, the energy of the charge-transfer exciton is close to that of local exciton when the interchain distance is lowered from 4.0 Å down to 3.0 Å. Besides, electron and hole couplings (off-diagonal terms) are also amplified in this strong interaction region. The distance dependences of the CT mixing in the lowest two excited states of OPV_n_, OT_n_ and OP_n_ are shown in Figure 6. There results indicate that CT contributions to the two low-lying excited states of those oligomer dimers increase with decreasing interchain distance.

The distance dependence of the CT contribution to the first excited state in the stilbene (OPV_2_) is consistent with the early INDO calculations by Cornil *et al.* [43]. These results might be related to some experimental data reported in the literature where the external pressure which changes the interchain distance can influence the quantum yield of conjugated systems [28]. The experimental results indicate that larger external pressure will dramatically decrease the emission intensity of the PPV [28]. Besides, we found that when the interchain distance falls into the range of 3.6-4.0 Å, the CT contribution of first excited states in those organic dimers is apparently large as shown in Figure 6. Note that the CT% of the first excited state of the oligomer pairs is slightly chain-length dependent. For instance, in the case of OPV_n_ with an interchain distance of 3.6 Å, this CT contribution of the first excited state drops from 26.9% when n = 2 to 23.1% for n = 7. For larger interchain separation, this effect is more significant. The trends of OT_n_ and OP_n_ are similar to OPV_n_, the only difference between those oligomer dimers is that the CT contributions of the first excited states of OT_n_ and OP_n_ are slightly higher and lower than that of OPV_n_, respectively. More interestingly, with increasing the chain size when the CT% reaches the minimum, the CT% contribution for the second excitation was suddenly increased and followed by a monotonic decrease. For instance, the jump points appear at OPV_5_ and OPV_6_ dimers with interchain distance by 3.6 and 3.8 Å, respectively. These results indicate that a new charge transfer contribution to the second excited state which is sensitive to the chain size and interchain distance. This new charge-transfer transitions that contributes to the second transition can be even larger than the CT mixing to the first transition. Based on the calculation on the dimer pairs, we found this behavior is quite general in the organic materials with larger conjugated length, and the charge-transfer contribution to the second excited state in a cofacial packing might be due to this contribution mainly. Analysis of the state function obtained by CM model provides a deeper insight into the origin for the CT contributions to the lowest two excited states in the oligomer dimers. We have examined the configuration-state function with interchain separation by 3.8 Å as shown in Table 2. In general, the major CT contribution to the first excited state is from HOMO to LUMO charge transfer but this contribution decreases with chain size [45,46]. Although transition energies of those oligomer dimers are quite different, the trends of the CT contributions in those dimers are quite similar; that is, the major CT contribution of the first excited state of smaller oligomer dimers is from HOMO to LUMO charge transfer and it decreases with chain size. For the second transition, the HOMO to LUMO charge transfer is the major CT contribution for shorter oligomer dimers. With increasing the chain size toward the long-chain limit, the major CT contribution is from two CT configurations which are HOMO - 1 to LUMO and HOMO to LUMO + 1 charge-transfer configuration-state functions.

**Table 2:** Calculated Electronic Transitions and State Functions for Cofacial polymer OPV_n_, OT_n_, OP_n_ Dimers Separated by 3.8 Å.

	Ε ^a^	CT%	CT-State function H_M1(M2)_→L_M2(M1)_ H-1 _M1(M2)_→L _M2(M1)_+ H _M1(M2)_→L++1 _M2(M1)_	
PPV2	3.70 (E_1_)	18.5	97.3%	
	4.15 (E_2_)	8.8	100.0%	
PPV7	2.80 (E_1_)	15.1	70.1%	
	3.02 (E_2_)	17.4		83.0%
TP3	2.70 (E_1_)	21.2	96.6%	
	3.23 (E_2_)	5.0	100.0%	
TP10	1.89 (E_1_)	16.8	74.4%	
	2.17 (E_2_)	20.3		82.9%
PPP3	3.68 (E_1_)	17.1	98.4%	
	4.08(E_2_)	6.4	100.0%	
PPP10	3.07 (E_1_)	13.8	70.1%	
	3.27 (E_2_)	15.7		82.5%

*^a^* Energy in eV.

## 4. Charge-Transfer Interactions in Cyclophanes

Cyclophane molecules are promising candidates for designing a new class of organic-based electro-optical devices because the chromophore-chromophore interactions with well defined distance and orientation can be easily controlled [112]. Recently, bichromophoric and multichromophoric cyclophanes have been studied for organic solids [113,114,115], biosensors [116,117], eletrocyclic reactions [118,119,120,121], two-photon absorptions [122,123,124], mixed-valence systems [125,126], and nonlinear optical materials [16,17,18]. Particularly, Bazan *et al.* have exploited the advantage of well-defined three-dimensional structures in cyclophanes that contains [2.2]paracyclophane core and found their potential applications in designing organic nonlinear optical materials [16,17]. More recently, it has been shown that chromophores with twisted π-electron systems in a cyclophane system having five-membered hetero-aromatic rings as electron donors and a bridging double bond as electron acceptor exhibit molecular hyperpolarizability with exceptionally high μβ values [18,127]. Interestingly, quantum-chemical calculations suggest that these cyclophane architectures with one or two unsaturated bridging double bonds reveal intramolecular charge transfer characteristics in the lowest excited state [18,127]. Accordingly, it is intriguing to study the nature of the excited states delocalization in detail for these bichromophoric cyclophanes with unsaturated tethers.

**Scheme 5:**
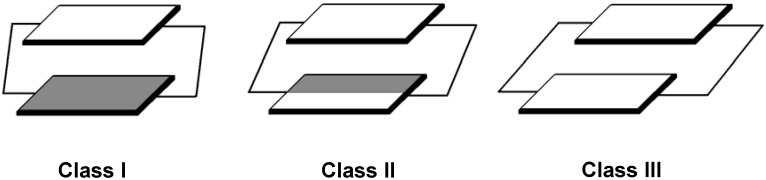
Three types of dimeric cyclophandienes with various dihedral angles between chromophores and tethered double bonds [47]. Reproduced with permission from American Chemical Society.

Here, we roughly distinguished the bichromophoric cyclophanes into three classes according to the dihedral angles between chromophores and the tethered double bonds as shown in Scheme 5. For molecules belonging to class I, the π-orbitals on the chromophore are almost perpendicular to π-orbitals on the tethers, and the pathway for this kind of excited state delocalization is clearly through-space. On the other hand, for molecules in class III, the π-orbitals of the chromophore are parallel to those of tethers and the delocalization pathway should be through-bond. The most interesting behavior appears in molecules belonging to the class II, where both kinds of delocalization pathways contribute to the transannular interaction between chromophores. It is an interesting synthetic challenge to construct cyclophane molecules belonging to the class II. Incorporation of five-membered heteroaromatic rings into teraryl-cyclophane system could lead to a dihedral angle between chromophore and tether of about 40-45 degrees. We have previously synthesized the furan-containing cyclophanes containing saturated and unsaturated tethers [18,127]. Cyclophanes with double-bonded tether could have a new low energy band in the absorption spectrum but the corresponding saturated cyclophane with only single bond tether did not. These results suggest that the three-dimensional cyclophene reveals a new charge resonance (CR) band. The delocalization pathway of the new CR band could come from through-bond and/or through-space delocalization. The main issue we want to address here is the delocalization pathway of the class II cyclophanes.

The main suggestion here is that the weight of the CT-exciton in the wavefunction can be used to determine the nature of delocalization (through-bond and/or through-space) in the cyclophane excitations. As discussed above, the widespread theoretical model, the supermolecular approach [54], in studying cyclophanes could not directly provide this critical information because of the inadequacy of the basis set based on completely delocalized molecular orbitals obtained by the Hartree-Fock calculations. CM calculation based on the construction of CI matrix with localized molecular orbitals is exploited to address the nature of excited states in the class II molecules. Furthermore, we also propose a simplified four-state CM model to extract electronic coupling from the calculation based on the supermolecular calculation. To the best of our knowledge, the nature of excited-state delocalization of cyclophane systems with a double-bonded tether has not been studied in detail; therefore, we try to find the influence of the π-bond tether on the CR band in the lowest-lying excited state by using the truncated CM method. As discussed above, the wavefunction of an excited state can be divided into two parts: local exciton and CT exciton contributions. The contribution of the CT exciton could be enhanced in double-layered cyclophane systems when the distances between two chromophores are close in space, and, therefore, this key information can be applied to determine the delocalization pathway by the CM method.

**Scheme 6:**
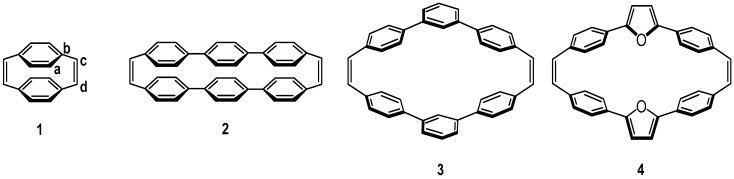
Molecular structures of cyclophandiene **1-4** [47]**.** Reproduced with permission from American Chemical Society.

The two benzene moieties in the AM1 optimized geometry of molecule **1** are almost lying in a near face-to-face arrangement with dihedral angle between the tethered double bond and the benzene moiety about 83° (C_a_C_b_ - C_c_C_d_ in Scheme 6). Hence, this molecule is an ideal model compound belonging to the class I. Similarly, the AM1-optimized geometry of molecule **2** also shows a slightly tilted cofacial arrangement of the two teraryl chromophores with a dihedral angle around 75°. Note that the aromatic rings in molecule **1** and **2** are both connected in para-linkage positions with the geometries belonging to the class I. On the other hand, the molecules with the meta-linkage have qualitatively different optimized geometries since their dihedral angles are reduced significantly. Hence this kind of molecules belongs to the class II. For instance, the AM1-optimized dihedral angle for molecule **3** is about 45°. In this regard, introducing meta-linkage aromatic moieties into cyclophanes may lead to molecular systems for studying class II models. Incorporation of five-membered heteroaromatics such as furan, thiophene and pyrrole can also be viewed as a particular case of meta-linkage cyclophane. The main difference between the meta-benzene and five-membered heteroaromatics is that the meta-benzene would interrupt the conjugation at two ends, while five-membered heteroaromatics remain efficient conjugation among them. In this article, the furan-containing [2.2]cyclophandiene **4** was employed as a model compound for class II. The AM1-optimized dihedral angle for this molecule is 45°, which is quite close to the value obtained from DFT-optimized geometry, 40°, indicating that the cyclophandiene **4** could be considered as class II molecule.

The nature of delocalization pathway in the lowest excited state of a cyclophane molecule can be rationalized by quantitatively studying the CT-exciton contribution of the corresponding excited state wavefunction in the CM representation. Here, we adopt a simple strategy to determine the relative importance of the through-bond and through-space contributions in cyclophane by comparing the weights of CT-excitons in the lowest excited state for the cyclophane under study and the hypothetical molecule with different fragmentation schemes as discussed in previous section. The results of calculations using the PPP and INDO Hamiltonians for excited states and the AM1 and DFT methods for the ground-state geometry are shown in Table 3. For the molecule **1** (entry 1 in Table 3), we can see that the contribution of the CT-exciton to the lowest excited state is close to that of the same molecule with the double-bond tethers removed. This can easily be understood since p-orbitals on tethers are almost perpendicular to those on benzene moieties. More careful examination shows that the weight of CT exciton diminishes slightly with the introduction of the tethers due to the increasing of conjugation length at chain end for the two chromophores in the cyclophane, and therefore the excitonic interaction is reduced. We could reasonably suggest that molecule **1** belongs to through-space case according to relative CT-exciton contributions. This is in agreement with the conclusions deduced from the NLO studies in which cyclophanes derived from the [2.2]paracyclophane are shown to be through-space delocalization [16,17].

**Table 3:** The relative contributions of CT-exciton to the lowest excited state of cyclophandiene **1-4** [47]. Reproduced with permission from American Chemical Society.

Entry	num (opt)	no tether (CT %)	tether (CT %)	T-space : T-bond (%)
1	**1** (AM1) *^a^*	27.79	27.04	100 : 0
2	**2** (AM1) *^a^*	6.32	5.22	100 : 0
3	**3** (AM1) *^a^*	0.14	19.65	1 : 99
4	**4** (AM1) *^a^*	0.99	4.41	22 : 78
5	**4** (DFT) *^a^*	0.60	6.85	9 : 91
6	**4** (AM1) *^b^*	0.74	2.51	29 : 71
7	**4** (DFT) *^b^*	0.46	4.87	9 : 91
8	**4** (AM1) *^c^*	0.65	3.12	21 : 79
9	**4** (DFT) *^c^*	0.36	4.52	8 : 92

*^a^* CM/PPP (Full SCI) model; *^b^* CM/PPP (Four State) model; *^c^* CM/INDO(S) (Four State) model.

For cyclophane **2** consisting of two para-connected teraryls with or without tethers removed as shown in the entry 2 of Table 3, the CT-exciton contributions to the lowest excited state decreased significantly due to the increasing chromophore length. Similar to the molecule **1**, through-space pathway is still the dominant channel for delocalization since this molecule also has the near cofacial arrangement. However, the molecule **3** with meta-connected moieties exhibits a significant CT-exciton contribution only when the tether was attached and, thus, this molecule can be regarded as belonging to the through-bond delocalization type. For molecule **4**, the contribution of CT-exciton for situation without tethers is also very small just like that of the molecule **3**, whereas the weight of CT-exciton can be enhanced almost five-fold by introducing the double-bond tethers. This means that the presence of tethers could significantly increase the electronic communication between two chromophores in the cyclophane. The calculated CT-exciton contribution based on the DFT-optimized geometry of molecule **4** shows a qualitatively similar result, except that the relative through-bond contribution is about 10% larger (entry 4) with respect to AM1 results (entry 5). This feature is owing to the smaller dihedral angle of the DFT geometry of **4**. Although these two structures are slightly different, the data indicate that molecule **4** is a case of through-bond delocalization.

**Figure 7:**
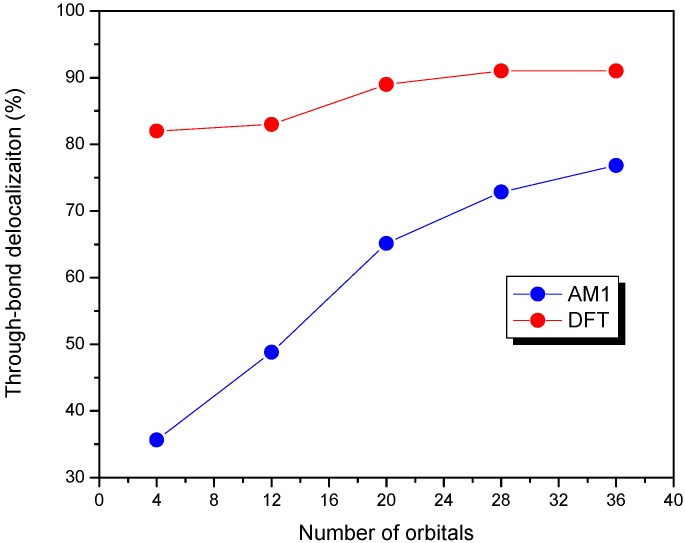
Various truncated CM model for molecule **4** with different optimized geometries for AM1 geometry (blue) and DFT geometry (red) [47]. Reproduced with permission from American Chemical Society.

We also examined the effect of the number of orbitals (*n*) included in the CM CI matrix on the nature of delocalization pathway for molecule **4** at two different optimized geometries using AM1 and DFT methods. The percentage of the through-bond delocalization for molecule **4** at the AM1-optimized geometry is quite sensitive to the number of orbitals included as shown in Figure 7. The through-bond contribution changes from 35% to 77% as the number of orbitals included is changed from 4 to 36. It is worth noting that, for the AM1 geometry, in the truncated model with only four orbitals, the through-bond contribution is smaller than that of through-space, whereas in the situation with more than twelve orbitals included, the through-bond channel becomes the dominant delocalization pathway. Alternatively, the through-bond contribution changes slightly from 82% to 91% with the increasing number of orbitals for the optimized geometry by the DFT method, which might be due to the fact that the DFT-optimized geometry shows a slightly planar structure (40°) than that by the AM1 calculation (45°).

**Figure 8:**
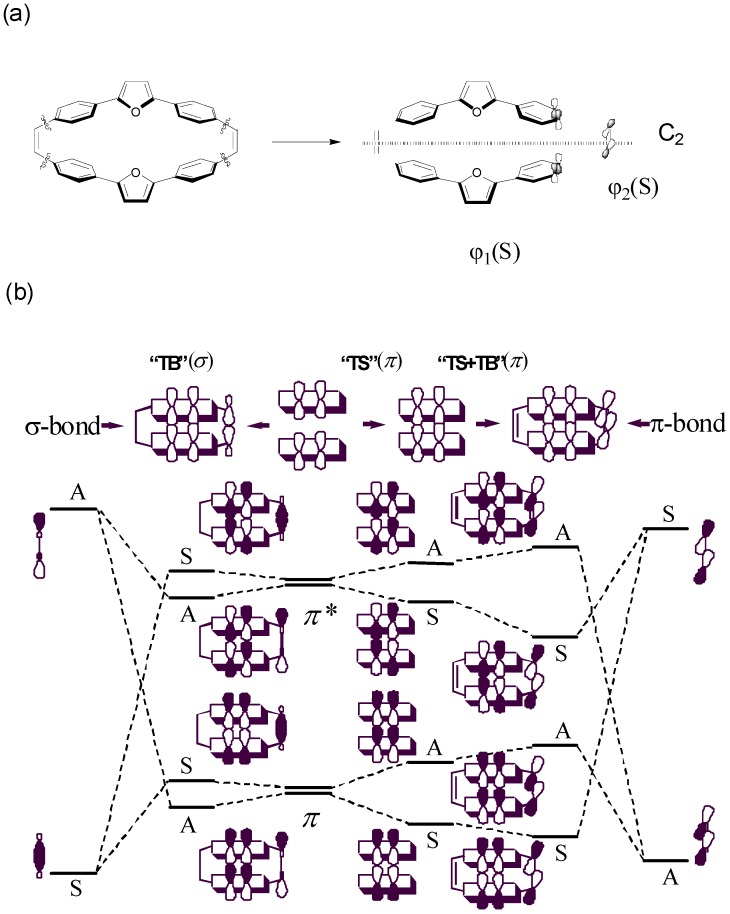
Orbital diagram for “through-space’’ and “through-bond’’ interaction in the class I/II cyclophandienes with C_2_ symmetry element [47]. Reproduced with permission from American Chemical Society.

We proposed a four-state CM model parameterized by supermolecular calculations such as PPP or INDO/S with SCI scheme. The critical information of the parameterized treatment other than SCI results is to obtain electron and hole transfer integrals by half of the splitting of the HOMO and LUMO levels of two monomers. Here, we discuss the orbital interaction by disconnection of the molecule **4** to two terayl chromophores and two tethers as given in Figure 8a. First, we consider two non-interacting identical teraryl monomers without tethering double bonds. In the absence of any through-space π-π interactions, two HOMOs (LUMOs) of the two monomers are degenerate in energy. When the two monomers do interact, the HOMOs (LUMOs) are split into two levels as shown in Figure 8b. In general, the splitting energy for HOMOs is generally larger than that of LUMOs which can be rationalized by the number nodal planes in these MOs. Therefore, through-space π-π interactions would lead to the hole transfer integral larger than electron transfer integral. In the following, the origin of the orbital interaction and molecular symmetry will be discussed. Since the molecule **4** belongs to the C_2h_ point group, the rotation about the two-fold axis, which interchange two fragment molecular orbitals, can be used to classify the delocalized molecular orbitals [128,129]. Now, we consider πorbitals on two teraryl chromophores. After operating the C_2_ rotation on the delocalized molecular orbital, the symmetry symbol of this orbital is **A** when the phase of the orbital changed and the other situation is **S**. Accordingly, the four frontier energy levels with through-space π-π interactions, are classified as **ASAS** symmetry in the order of descending energy. Second, we include the tether portions, and it is reasonable to single out the essential σ and π orbitals without considering the σ bonds between tethers and chromophores [128,129]. The symmetries of the HOMO and LUMO of the π bond in the tether are **A** and **S**, respectively. According to the symmetry constraint, the HOMO (**A**) of the π bond in the tether would interact with the HOMO (**A**) and LUMO + 1 (**A**) of the chormophore portion and also the LUMO (**S**) of the π bond in the tether can interact with the LUMO (**S**) and HOMO-1 (**S**) of the chromophroes. It is noteworthy that the **SS** π-π interaction is bonding/constructive as shown in Figure 8b. According to perturbation theory, the **SS** interaction between LUMO of the π-bond tethers and the LUMO of the chrompores would be larger than that of HOMO-1 because of energy difference of the energy levels. As a result, the splitting of the LUMO and LUMO + 1 would be enhanced by through-bond (π) interaction. Note that after allowing through-bond π−π interaction, the energy levels would remain the same symmetry order as those of through-space. Therefore, both through-space (π−π) and though-bond (π−π) interaction will increase the splitting energy and this character might be used for the estimation of the electron and hole transfer integrals. Otherwise, if any, the through-bond (σ−π) interaction is considered, the symmetry symbols of the sigma orbitals are **SASA** in the order of descending energy. This feature may reduce the estimated transfer integrals, and the CM four-state model would not work well when the σ−π interaction is significant because of underestimation of the transfer integrals. The truncated four-state model provide a reasonably connection compared with CM/PPP (Full SCI) as shown in Table 3. The parameters for the four-state model is based on PPP and INDO/S Hamiltonian with a SCI scheme, and both the AM1 and DFT geometries of **4** show reasonable relative CT-excitons (entry 6-9 in Table 3). These results indicate the nature of the delocalization in the lowest excited state of molecule **4** is mainly due to through-bond pathway. This truncated model gives a good agreement with CM/PPP (Full SCI) method which is according to correctly estimate electron and hole transfer integrals. The CM/INDO(S) four state model gives consistent results compared with CM/PPP (Full SCI) method which indicates that the through-bond (σ−π) contribution to the excited state is negligible in molecule **4**. It is noteworthy that the comparable results of CM/INDO(S) and CM/PPP (Full SCI) calculations are cross reference. In other words, the π-based CM/PPP (Full SCI) model as well as the truncated four-state model might be good enough for determining the delocalization pathway in the lowest excited state.

The measurement and interpretation of the electronic spectra of biphenylene have been extensively studied throughout last few decades. Recently, biphenylene-based cyclophane **5** has been synthesized by Leung *et al.* [51].

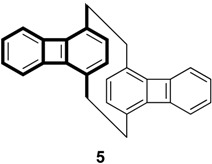



In our theoretical treatment, we mimic the electronic properties of **5** by a model with two biphenylene units in close contact. On the basis of the AM1-optimized biphenylene geometry, we compute excited state properties of the cofacial (H-type) and head-to-tail (J-type) dimers by model 1. The stackings of the biphenylene units in the form of *H*-aggregation and in the form of *J*-aggregation are then compared.

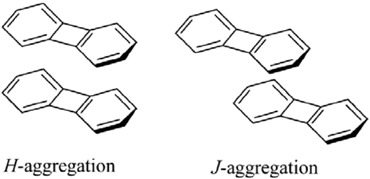



**Figure 9:**
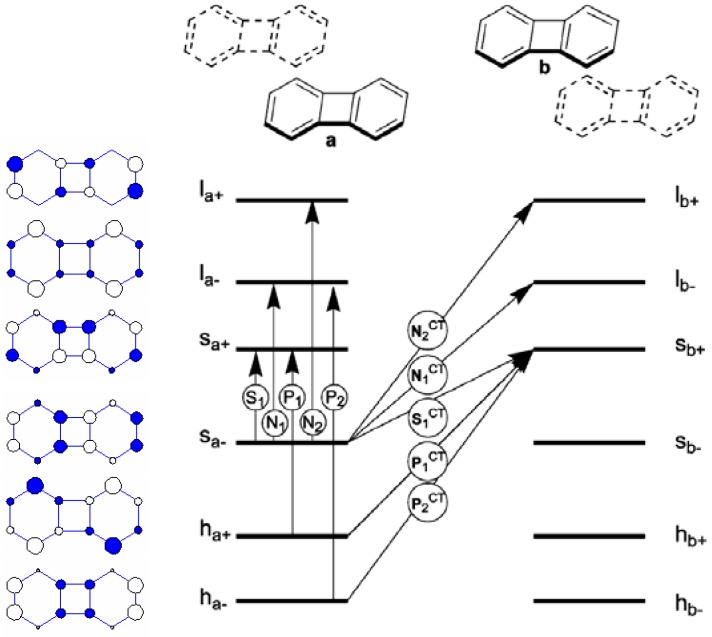
Hückel MO’s of the J-aggregated biphenylenes, their energies, perimeter labels, and the five singly excited configurations responsible for the S_1_, N_1_, N_2_, P_1_, and P_2_ states. [51]. Reproduced with permission from American Chemical Society.

The nature of electronic excited states of cyclophane consisting of two cyclic π-electron systems derived from a 4N-electron perimeter is quite different from those systems based on a (4N + 2)-electron perimeter. To explore the effect of the transannular interaction on the most important low-lying electronic excited states, particularly, the relative weights of local exciton and chargetransfer exciton, we have adopted the composite-molecule method of Longuet-Higgins and Murrel [55] to perform configuration interaction (model 1). Two types of biphenylene cyclophanes and their face-to-face (H-type) and head-to-tail (J-type) arrangements are investigated. The perpendicular distance between two plarnar biphenylenes is chosen to be within 3.0-4.5 Å. For a qualitative discussion, only a situation with *d* = 4.0 Å is reported. To facilitate the discussion, we show a schematic diagram of six local perimeter molecular orbitals and the corresponding energy diagram of a monomeric biphenylene in Figure 9. There are two types of electronic excitations: those excitations involve the creation of local exciton and those configurations with charge transfer between two moieties in the cyclophane. The notation and classification of molecular orbitals and electronic states closely follow the perimeter labels proposed by Fleischhauer *et al.* [73] Only five (out of 10) essential local exciton states and five (out of 10) charge transfer configurations are shown in Figure 9.

**Figure 10:**
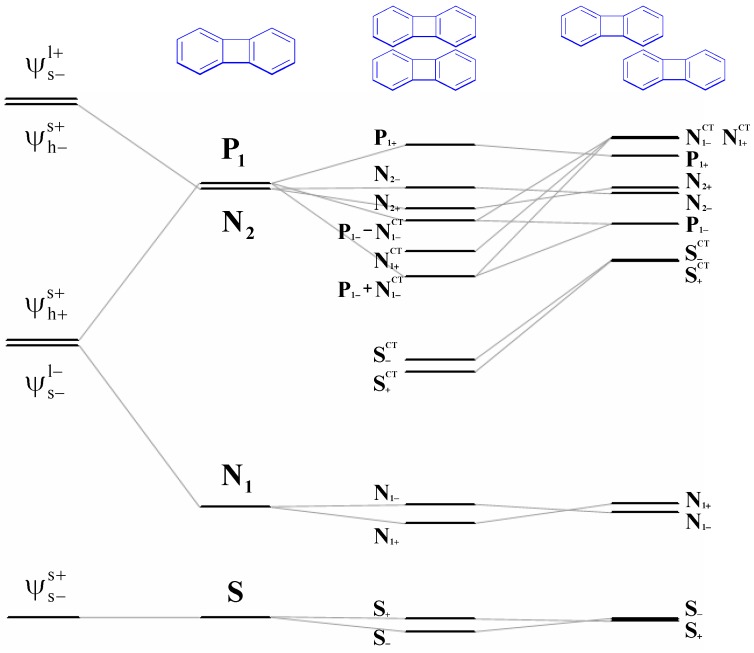
State correlation diagrams of biphenylene, H-aggregrated biphenylene dimer (d = 4 Å), and J-aggregrated biphenylene dimer (d = 4 Å). [51]. Reproduced with permission from American Chemical Society.

The results of calculations for the biphenylene cyclophane in the two different spatial H-type and J-type aggregations are shown in Table 4 and Table 5. The state correlation diagram of these two states between monomer and two types of cyclophanes is shown in Figure 10. The lowest two electronic states of the H-aggregated and the J-aggregated biphyenylenes (states 1 and 2 in Table 4 and Table 5) originate from symmetric and antisymmetric linear combinations of monomeric s_–_→ s_+_ local transitions. Both states have vanishing oscillator strength as expected from the null value of corresponding monomeric transitions. The experimental observations of weak fluorescence progression around 530-550 nm are probably due to the vibronic borrowing effect [51]. In addition, the larger overlap between two moieties in the H-type cyclophane with face-to-face arrangement leads to a small mixing of charge-transfer configuration. The exciton coupling between two moieties estimated from the energy splitting between these two states ranges from 0.1 eV (J-cyclophane) to 0.5 eV (H-cyclophane). With such a small exciton coupling, we expect that these two low-lying electronic states exhibit trapped localized excitation in the presence of exciton-phonon coupling.

The next two electronic states (states 3 and 4 in Table 4 and Table 5) have their origin as s_–_ → l_–_ local excitations. These two states consist mostly of delocalized neutral exciton states with almost no charge transfer in the J-type cyclophane with two moieties in head-to-tail alignment. The state with in-phase oscillation of transition dipole of two local excitons (N_1+_ in J-type and N_1-_ in H-type cyclophane) generates small absorption intensity. Hence, we believe that the observed minimal peak of around 370 nm in the experiment is due to this excitation [51]. States 5 and 6 are two degenerate dark electronic states involving symmetric and antisymmetric combinations of charge-transfer configurations from s_1-_ → s_2+_ and s_2-_ → s_1+_. The two electronic states originating from two local P_1_ excitations (states 7 and 10 in J-cyclophane; and states 7 and 12 in H-cyclophane) have the largest exciton splittings of 2.56 and 4.91 eV, respectively (Figure 10). This indicates that there exists a strong dipole-dipole coupling between these two moieties. For both arrangements, the delocalized exciton with higher energy carries most of the intensity, which is in agreement with the experimental observation that an intensive absorption around 260 nm is seen. Interestingly, this brightest state of the J-aggregated biphenylenes contains 5% of CT characteristics while the H-aggregated biphenylenes contains 11% of the CT characteristic. This kind of excimeric character of the excited states becomes even more dramatic when the distance between two moieties is closer, for instance, CT% = 50% when *d* = 3.5 Å in the case of H-type cyclophane. Hidden behind the strong absorption band due to P_1+_, there are several forbidden and weakly allowed electronic states originating from N_2_ and N_1_^CT^ local states. 

**Table 4:** Calculated Electronic Transitions for [2,2]Biphenylenophane in H-Aggregation, the Face-to-Face Arrangement. [51]. Reproduced with permission from American Chemical Society.

State	E*^a^*	f	state function
1	26.96	0	0.69$S_{1-}$ + 0.15$S_{1-}^{CT}$
2	27.46	0	0.70$S_{1-}$
3	31.02	0	0.69$N_{1+}$ + 0.12$N_{1-}^{CT}$
4	31.71	0.19	0.70$N_{1-}$
5	36.67	0	0.70$S_{1+}^{CT}$
6	37.09	0	0.69$S_{1-}^{CT}$ + 0.15$S_{1-}$
7	40.24	0	0.52$P_{1-}$ + 0.44$N_{1-}^{CT}$ + 0.18$P_{1-}^{CT}$
8	41.19	0.03	0.70$N_{1+}^{CT}$
9	42.33	0	0.54$N_{1-}^{CT}$- 0.42$P_{1-}$- 0.14$P_{1-}^{CT}$
10	42.76	0	0.68$N_{2+}$ - 0.19$N_{2-}^{CT}$
11	43.54	0.22	0.70$N_{2-}$ + 0.11$N_{2+}^{CT}$
12	45.15	3.60	0.66$P_{1+}$ - 0.24$P_{1+}^{CT}$

*^a^* Energy in 10^3^ cm^-1^.

**Table 5:** Calculated Electronic Transitions for [2,2]Biphenylenophane in J-Aggregation, the Head-to-Tail Arrangement.*^a^* [51]. Reproduced with permission from American Chemical Society.

State	E	f	state function
1	27.40	0	0.70$S_{1+}$
2	27.51	0	0.70$S_{1-}$
3	31.45	0	0.70$N_{1-}$
4	31.77	0.23	0.70$N_{1+}$
5	40.79	0	0.70$S_{1+}^{CT}$
6	40.80	0	0.70$S_{1-}^{CT}$
7	42.22	0	0.70$P_{1-}$
8	43.38	0.04	0.70$N_{2-}$
9	43.59	0	0.70$N_{2+}$
10	44.78	3.84	0.68$P_{1+}$ - 0.16$N_{1+}^{CT}$
11	45.42	0	0.70$N_{1-}^{CT}$
12	45.46	0.24	0.68$N_{1+}^{CT}$ + 0.16$P_{1+}$

*^a^* Energy in 10^3^ cm^-1^.

## 5. Synopsis

The purpose of this review was to illustrate that quantum-chemical calculations can provide a good insight into the excited-state electronic structures of functional organic materials in the solid state. It is clear that the composite-molecule approach gives a better understanding of the charge-transfer interaction in the excited state compared to supermolecular approach and represents a significant improvement with respect to the traditional exciton theories that are usually used to predict the optical properties of interacting chains. Furthermore, we also demonstrated that this method can be used to address the pathway of the delocalization (through-bond and/or through-space) in the lowest excited state for cyclophanes by combining the charge-transfer contributions calculated on the cyclophanes and the corresponding hypothetical molecules with tethers removed. This review represents a step forward in the understanding of the nature of the charge-transfer interactions in the excited state of organic functional materials.

## References

[1] Mitschke U., Bäuerle P. (2000). The electroluminescence of organic materials. J. Mater. Chem..

[2] Hreha R.D., George C.P., Haldi A., Domercq B., Malagoli M., Barlow S., Brédas J.-L., Kippelen B., Marder S.R. (2003). 2,7-Bis(diarylamino)-9,9-dimethylfluorenes as Hole-Transport Materials for Organic Light-Emitting Diodes. Adv. Funct. Mater..

[3] Ahn J.H., Wang C., Perepichka I.F., Bryce M.R., Petty M.C. (2007). Blue organic light emitting devices with improved colour purity and efficiency through blending of poly(9,9-dioctyl-2,7-fluorene) with an electron transporting material. J. Mater. Chem..

[4] Shaheen S.E., Jabbour G.E., Kippelen B., Peyghambarian N., Anderson J.D., Marder S.R., Armstrong N.R., Bellmann E., Grubbs R.H. (1999). Organic light-emitting diode with 20 lm/W efficiency using a triphenyldiamine side-group polymer as the hole transport layer. Appl. Phys. Lett..

[5] Miyata S., Nalwa H.S. (1998). Organic Electroluminescence Materials and Devices.

[6] de Bettignies R., Nicolas Y., Blanchard P., Levillain E., Nunzi J.M., Roncali J. (2003). Planarized Star-Shaped Oligothiophenes as a New Class of Organic Semiconductors for Heterojunction Solar Cells. Adv. Mater..

[7] Pei Q., Yang Y., Yu G., Zhang Ch., Heeger A. J. (1996). Polymer Light-Emitting Electrochemical Cells: *In situ* Formation of a Light-Emitting p-n Junction. J. Am. Chem. Soc..

[8] Zhan X., Tan Z., Domercq B., An Z., Zhang X., Barlow S., Li Y., Zhu D., Kippelen B., Marder S.R. (2007). A High-Mobility Electron-Transport Polymer with Broad Absorption and Its Use in Field-Effect Transistors and All-Polymer Solar Cells. J. Am. Chem. Soc..

[9] Ray A., Goswami D., Chattopadhyay S., Bhattacharya S. (2008). Photophysical and Theoretical Investigations on Fullerene/Phthalocyanine Supramolecular Complexes. J. Phys. Chem. A.

[10] Akaike K., Kanai K., Ouchi Y., Seki K. (2010). Impact of Ground-State Charge Transfer and Polarization Energy Change on Energy Band Offsets at Donor/Acceptor Interface in Organic Photovoltaics. Adv. Funct. Mater..

[11] Li Y.-X., Tao X.-T., Wang F.-J., He T., Zhang L.-L., Jiang M.-H. (2009). Effect of substituents on the properties of distyrylarylene-based field-effect transistor materials. Chem. Phys. Lett..

[12] Ramajothi J., Ochiai S., Kojima K., Mizutani T. (2008). Performance of Organic Field-Effect Transistor Based on Poly(3-hexylthiophene) as a Semiconductor and Titanium Dioxide Gate Dielectrics by the Solution Process. Jpn. J. Appl. Phys..

[13] Yasuda T., Saito M., Nakamura H., Tsutsui T. (2006). Organic Field-Effect Transistors Based on Oligo-p-Phenylenevinylene Derivatives. Jpn. J. Appl. Phys..

[14] Haddock J.N., Zhang X., Zheng S., Zhang Q., Marder S.R., Kippelen B. (2006). A comprehensive study of short channel effects in organic field-effect transistors. Org. Electron..

[15] Kim P., Zhang X.-H., Domercq B., Jones S.C., Hotchkiss P.J., Marder S.R., Kippelen B., Perry J.W. (2008). Solution-processible high-permittivity nanocomposite gate insulators for organic field-effect transistors. Appl. Phys. Lett..

[16] Bartholomew G.P., Ledoux I., Mukamel S., Bazan G.C., Zyss J. (2002). Three-Dimensional Nonlinear Optical Chromophores Based on Through-Space Delocalization. J. Am. Chem. Soc..

[17] Zyss J., Ledoux I., Volkov S., Chernyak V., Mukamel S., Bartholomew G.P., Bazan G.C. (2000). Through-Space Charge Transfer and Nonlinear Optical Properties of Substituted Paracyclophane. J. Am. Chem. Soc..

[18] Lin H.-C., Lin W.-Y., Bai H.-T., Chen J.-H., Jin B.-Y., Luh T.-Y. (2007). A Bridging Double Bond as an Electron Acceptor for Optical Nonlinearity of Furan-Containing [n.2]Cyclophenes. Angew. Chem. Int. Ed..

[19] Holt J., Singh S., Drori T., Zhang Y., Vardeny Z.V. (2009). Optical probes of π-conjugated polymer blends with strong acceptor molecules. Phys. Rev. B.

[20] Suresh P., Sharma S.K., Roy M.S., Sharma G.D. (2009). Photocurrent mechanism and photovoltaic properties of photo-electrochemical device based on PPAT and PPAT:TY blend. Synth. Met..

[21] Yin C., Schubert M., Bange S., Stiller B., Castellani M., Neher D., Kumke M., Hörhold H.-H. (2008). Tuning of the Excited-State Properties and Photovoltaic Performance in PPV-Based Polymer Blends. J. Phys. Chem. C.

[22] Lee J.Y., Bhattacharya B., Kim D.-W., Park J.-K. (2008). Poly(ethylene oxide)/Poly(dimethylsiloxane) Blend Solid Polymer Electrolyte and Its Dye-Sensitized Solar Cell Applications. J. Phys. Chem. C.

[23] Li G., Shrotriya V., Yao Y., Huang J., Yang Y. (2007). Manipulating regioregular poly(3-hexylthiophene) : [6,6]-phenyl-C_61_-butyric acid methyl ester blends-route towards high efficiency polymer solar cells. J. Mater. Chem..

[24] Sariciftci N.S., Smilowitz L., Heeger A.J., Wudl F. (1992). Photoinduced Electron Transfer from a Conducting Polymer to Buckminsterfullerene. Science.

[25] Halls J.J.M., Walsh C.A., Greenham N.C., Marseglia E.A., Friend R.H., Moratti S.C., Holmes A.B. (1995). Efficient photodiodes from interpenetrating polymer networks. Nature.

[26] Granström M., Petrisch K., Arias A.C., Lux A., Andersson M.R., Friend R.H. (1998). Laminated fabrication of polymeric photovoltaic diodes. Nature.

[27] Yu G., Wang J., McElvain J., Heeger A.J. (1998). Large-Area, Full-Color Image Sensors Made with Semiconducting Polymers. Adv. Mater..

[28] Webster S., Batchelder D.N. (1996). Absorption, luminescence and Raman spectroscopy of poly(p-phenylene vinylene) at high pressure. Polymer.

[29] Kippelen B., Brédas J.-L. (2009). Organic photovoltaics. Energy Environ. Sci..

[30] Conwell E.M., Perlstein J., Shaik S. (1996). Interchain photoluminescence in poly(phenylene vinylene) derivatives. Phys. Rev. B.

[31] Bazan G.C., Oldham W.J., Lachicotte R.J., Tretiak S., Chernyak V., Mukamel S. (1998). Stilbenoid Dimers: Dissection of a Paracyclophane Chromophore. J. Am. Chem. Soc..

[32] Clark A. E., Qin C. Y., Li A. D. Q. (2007). Beyond Exciton Theory: A Time-Dependent DFT and Franck−Condon Study of Perylene Diimide and Its Chromophoric Dimer. J. Am. Chem. Soc..

[33] Kuhlman T. S., Lemke H. T., Sølling T. I., Velardez G. F., Henriksen N. E., Møller K. B. (2009). Comment on “Theoretical Investigation of Perylene Dimers and Excimers and Their Signatures in X-Ray Diffraction”. J. Phys. Chem. A.

[34] Dreuw A., Head-Gordon M. (2004). Failure of Time-Dependent Density Functional Theory for Long-Range Charge-Transfer Excited States: The Zincbacteriochlorin−Bacteriochlorin and Bacteriochlorophyll−Spheroidene Complexes. J. Am. Chem. Soc..

[35] Pabst M., Köhn A. (2010). Excited States of [3.3](4,4’)Biphenylophane: The Role of Charge-Transfer Excitations in Dimers With π-π Interaction. J. Phys. Chem. A.

[36] Pope M., Swenberg C. (1982). Electronic Processes in Organic Materials.

[37] Pope M., Swenberg C. (1999). Electronic Processes in Organic Crystals and Polymers.

[38] Harrison N.T., Hayes G.R., Philipps R.T., Friend R.H. (1996). Singlet Intrachain Exciton Generation and Decay in Poly(p-phenylenevinylene). Phys. Rev. Lett..

[39] Frolov S.V., Gellermann W., Vardeny Z.V., Ozaki M., Yoshino K. (1997). Picosecond photophysics of luminescent conducting polymers from excitons to polaron pairs. Synth. Met..

[40] Rothberg L.J., Yan M., Papadimitrakopoulos F., Galvin M.E., Kwock E.K., Miller T.M. (1996). Photophysics of phenylenevinylene polymers. Synth. Met..

[41] Papadimitrakopoulos F., Konstadinidis K., Miller T.M., Opila R., Chandross E.A., Galvin M.E. (1994). The Role of Carbonyl Groups in the Photoluminescence of Poly(p-phenylenevinylene). Chem. Mater..

[42] Jenekhe S.A., Osaheni J.A. (1994). Excimers and Exciplexes of Conjugated Polymers. Science.

[43] Cornil J., dos Santos D.A., Crispin X., Silbey R., Brédas J.-L. (1998). Influence of Interchain Interactions on the Absorption and Luminescence of Conjugated Oligomers and Polymers: A Quantum-Chemical Characterization. J. Am. Chem. Soc..

[44] Tretiak S., Saxena A., Martin R.L., Bishop A.R. (2000). Interchain Electronic Excitations in Poly(phenylenevinylene) (PPV) Aggregates. J. Phys. Chem. B.

[45] Yeh M.-Y., Lin H.-C., Lee S.-L., Chen C.-h., Lim T.-S., Fann W.-S., Luh T.-Y. (2007). Thorpe-Ingold Effect on the Conformation and Photophysical Properties of Dialkylsilylene-Spaced Divinylarene Copolymers. Chem. Commun..

[46] Lin H.-C., Jin B.-Y. (2010). Interchain Interactions in Organic Conjugated Dimers: A Composite-Molecule Approach. J. Phys. Chem. A.

[47] Lin H.-C., Jin B.-Y. (2008). Three-Dimensional Through-Space/Through-Bond Delocalization in Cyclophane Systems: A Molecule-in-Molecule Approach. J. Phys. Chem. A.

[48] Jin B.-Y., Silbey R. (1995). Lattice relaxation in the 1B_u_ state for the finite polyenes. J. Chem. Phys..

[49] Orlandi G., Zerbetto F., Zgierski M.Z. (1991). Theoretical analysis of spectra of short polyenes. Chem. Rev..

[50] You Z.-Q., Hsu C.-P., Fleming G.R. (2006). Triplet-triplet energy-transfer coupling: Theory and calculation. J. Chem. Phys..

[51] Leung M.-K., Viswanath M.B., Chou P.-T., Pu S.-C., Lin H.-C., Jin B.-Y. (2005). The Phane Properties of anti-[2.2](1,4)Biphenylenophane. J. Org. Chem..

[52] Howard I.A., Zutterman F., Deroover G., Lamoen D., Van Alsenoy C. (2004). Approaches to Calculation of Exciton Interaction Energies for a Molecular Dimer. J. Phys. Chem. B.

[53] Gierschner J., Huang Y.-S., Van Averbeke B., Cornil J., Friend R. H., Beljonne D. (2009). Excitonic *versus* electronic couplings in molecular assemblies: The importance of non-nearest neighbor interactions. J. Chem. Phys..

[54] Beljonne D., Cornil J., Silbey R., Millié P., Brédas J.L. (2000). Interchain interactions in conjugated materials: The exciton model *versus* the supermolecular approach. J. Chem. Phys..

[55] Longuet-Higgins H.C., Murrell J.N. (1955). The Electronic Spectra of aromatic mplecules.5. The Interaction of 2 conjugated systems. Proc. Phys. Soc. A..

[56] Warshel A., Parson W.W. (1987). Spectroscopic properties of photosynthetic reaction centers. 1. Theory. J. Am. Chem. Soc..

[57] Parson W.W., Creighton S., Warshel A. (1989). Calculations of charge-transfer transition energies and spectroscopic properties of a molecular crystal: methylbacteriopheophorbide. J. Am. Chem. Soc..

[58] Harcourt R.D., Scholes G.D., Ghiggino K.P. (1994). Rate expressions for excitation transfer. II. Electronic considerations of direct and through–configuration exciton resonance interactions. J. Chem. Phys..

[59] Hennebicq E., Pourtois G., Scholes G.D., Herz L.M., Russell D.M., Silva C., Setayesh S., Grimsdale A.C., Mullen K., Bredas J.-L., Beljonne D. (2005). Exciton Migration in Rigid-Rod Conjugated Polymers: An Improved Förster Model. J. Am. Chem. Soc..

[60] Szabo A., Ostlund W.S. (1982). Modern Quantum Chemistry: Introduction to Advanced Electronic Structure Theory.

[61] Balzani V. (2001). Electron Transfer in Chemistry.

[62] Bixon M., Jortner J. (1999). Electron Transfer: From Isolated Molecules to Biomolecules.

[63] Newton M.D. (1991). Quantum chemical probes of electron-transfer kinetics: the nature of donor-acceptor interactions. Chem. Rev..

[64] Brédas J.L., Beljonne D., Coropceanu V., Cornil J. (2004). Charge-Transfer and Energy-Transfer Processes in π-Conjugated Oligomers and Polymers: A Molecular Picture. Chem. Rev..

[65] Li X.Y., Tang X.S., He F.C. (1999). Electron transfer in poly(p-phenylene) oligomers: effect of external electric field and application of Koopmans. Chem. Phys..

[66] Li X.Y., He F.C. (1999). Electron transfer between biphenyl and biphenyl anion radicals: Reorganization energies and electron transfer matrixe. J. Comput. Chem..

[67] Coropceanu V., Cornil J., Da Silva Filho D.A., Olivier Y., Silbey R., Brédas J.L. (2007). Charge Transport in Organic Semiconductors. Chem. Rev..

[68] Blancafort L., Voityuk A.A. (2006). CASSCF/CAS-PT2 Study of Hole Transfer in Stacked DNA Nucleobases. J. Phys. Chem. A.

[69] Pati R., Karna S.P. (2001). *Ab initio* Hartree-Fock study of electron transfer in organic molecules. J. Chem. Phys..

[70] RodriguezMonge L., Larsson S. (1996). Conductivity in Polyacetylene. 3. *Ab initio* Calculations for a Two-Site Model for Electron Transfer. J. Phys. Chem..

[71] Dewar M.J.S., Zoebisch E.G., Healy E.F., Stewart J.J.P. (1985). Development and use of quantum mechanical molecular models. 76. AM1: a new general purpose quantum mechanical molecular model. J. Am. Chem. Soc..

[72] Parr R.G. (1963). The Quantum Theory of Molecular Electronic Structure.

[73] Fleischhauer J, Ho1weler U., Spanget-Larsen J., Raabe G., Michl J. (2004). Magnetic Circular Dichroism of Nonaromatic Cyclic π-Electron Systems. 5. Biphenylene and Its Aza Analogues. J. Phys. Chem. A..

[74] Kirtman B., Dykstra C.E., Champagne B. (1999). Major intermolecular effects on nonlinear electrical response in a hexatriene model of solid state polyacetylene. Chem. Phys. Lett..

[75] Guillaume M., Champagne B. (2005). Modeling the electric field third-order nonlinear responses of an infinite aggregate of hexatriene chains using the electrostatic interaction model. Phys. Chem. Chem. Phys..

[76] Brédas J.-L., Silbey R. (2009). CHEMISTRY: Excitons Surf Along Conjugated Polymer Chains. Science.

[77] Paci I., Johnson J.C., Chen X., Rana G., Popovic D., David D.E., Nozik A.J., Ratner M.R., Michl J. (2006). Singlet fission for dye-sensitized solar cells: Can a suitable sensitizer be found?. J. Am. Chem. Soc..

[78] Castellan A., Michl J. (1978). Magnetic circular-dichroism of cyclic pi-electron systems. 4. Aza analogs of benzene. J. Am. Chem. Soc..

[79] Pople J.A., Beveridge D.L., Dobosh P.A. (1967). Approximate Self-Consistent Molecular-Orbital Theory. V. Intermediate Neglect of Differential Overlap. J. Chem. Phys..

[80] Zerner M.C., Loew G.H., Kichner R.F., Mueller-Westerhoff U.T. (1980). An intermediate neglect of differential overlap technique for spectroscopy of transition-metal complexes. Ferrocene. J. Am. Chem. Soc..

[81] Cornil J., Vanderdonckt S., Lazzaroni R., dos Santos D.A., Thys G., Geise H.J., Yu L.-M., Szablewski M., Bloor D., Lögdlund M., Salaneck W.R., Gruhn N.E., Lichtenberger D.L., Lee P.A., Armstrong N.R., Brédas J.L. (1999). Valence Electronic Structure of π-Conjugated Materials: Simulation of the Ultraviolet Photoelectron Spectra with Semiempirical Hartree-Fock Approaches. Chem. Mater..

[82] Lofthagen M., Chadha R., Siegel J.S. (1991). Synthesis, structures, and dynamics of a macrocyclophane. J. Am. Chem. Soc..

[83] Tobe Y., Kawaguchi M., Kakiuchi K., Naemura K. (1993). [2.2]Orthoparacyclophane: the last and most strained [2.2] cyclophane. J. Am. Chem. Soc..

[84] Srinivasan M., Sankararaman S., Dix I., Jones P.G. (2000). Synthesis and Structure of a New [6.6]Metacyclophane with Enediyne Bridges. Org. Lett..

[85] Sancho-García J.C., Pérez-Jiménez A.J. (2009). Assessment of double-hybrid energy functionals for π conjugated systems. J. Chem. Phys..

[86] Lee C., Yang W., Parr R.G. (1988). Development of the Colle-Salvetti correlation-energy formula into a functional of the electron density. Phys. Rev. B.

[87] Becke A.D. (1988). Density-functional exchange-energy approximation with correct asymptotic behavior. Phys. Rev. A.

[88] Becke A.D. (1993). Density-functional thermochemistry. III. The role of exact exchange. J. Chem. Phys..

[89] Ditchfield R., Hehre W.J., Pople J.A. (1971). Self-Consistent Molecular-Orbital Methods. IX. An Extended Gaussian-Type Basis for Molecular-Orbital Studies of Organic Molecules. J. Chem. Phys..

[90] Hehre W.J., Ditchfield R., Pople J.A. (1972). Self-Consistent Molecular Orbital Methods. XII. Further Extensions of Gaussian-Type Basis Sets for Use in Molecular Orbital Studies of Organic Molecules. J. Chem. Phys..

[91] Hariharan P.C., Pople J.A. (1974). Accuracy of AHn equilibrium geometries by single determinant molecular orbital theory. Mol. Phys..

[92] Gordon M.S. (1980). The isomers of silacyclopropane. Chem. Phys. Lett..

[93] Hariharan P.C., Pople J.A. (1973). Influence of polarization functions on molecular-orbital hydrogenation energies. Theor. Chim. Acta.

[94] Walden S., Glatzhofer D.T. (1997). Distinctive Normal Harmonic Vibrations of [2.2]Paracyclophane. J. Phys. Chem. A.

[95] Hensler D., Hohlneicher G. (1998). Theoretical Study on the Molecular Distortions in [2.2]Paracyclophane and Cyclobutane. J. Phys. Chem. A.

[96] Zojer E., Cornil J., Leising G., Brédas J.L. (1999). Theoretical investigation of the geometric and optical properties of neutral and charged oligophenylenes. Phy. Rev. B.

[97] McIntire M.J., Manas E.S., Spano F.C. (1997). Spontaneous emission and absorption in model aggregates of π-conjugated oligomers. J. Chem. Phys..

[98] Manas E.S., Spano F.C. (1998). Absorption and spontaneous emission in aggregates of conjugated polymers. J. Chem. Phys..

[99] Van Vooren A., Lemaur V., Ye A., Beljonne D., Cornil J. (2007). Impact of Bridging Units on the Dynamics of Photoinduced Charge Generation and Charge Recombination in Donor-Acceptor Dyads. ChemPhysChem.

[100] Cornil J., Beljonne D., Calbert J.-P., Brédas J.L. (2001). Interchain Interactions in Organic π-Conjugated Materials: Impact on Electronic Structure, Optical Response, and Charge Transport. Adv. Mater..

[101] Koh S.E., Risko C., da Silva Filho D.A., Kwon O., Facchetti A., Brédas J.L., Marks T.J., Ratner M.A. (2008). Modeling Electron and Hole Transport in Fluoroarene-Oligothiopene Semiconductors: Investigation of Geometric and Electronic Structure Properties. Adv. Funct. Mater..

[102] Valeev E.F., Coropceanu V., da Silva Filho D.A., Salman S., Brédas J.L. (2006). Effect of Electronic Polarization on Charge-Transport Parameters in Molecular Organic Semiconductors. J. Am. Chem. Soc..

[103] Marcelli A., Foggi P., Moroni L., Gellini C., Salvi P.R., Badovinac I.J. (2007). Relaxation Properties of Porphyrin, Diprotonated Porphyrin, and Isoelectronic Tetraoxaporphyrin Dication in the S2 State. J. Phys. Chem. A.

[104] Susumu K., Kunimoto K., Segawa H., Shimidzu T. (1995). Relaxation Process of the Singlet Excited State of "Wheel-and-Axle-Type" Phosphorus(V) Porphyrin Dimers. J. Phys. Chem..

[105] Susumu K., Kunimoto K., Segawa H., Shimidzu T. (1995). Control of photophysical properties of “wheel-and-axle-type” phosphorus (V) porphyrin dimers by electronic symmetry breaking Journal of Photochemistry. J. Photochem. Photobiol. A: Chemistry.

[106] Willert A., Bachilo S., Rempel U., Shulga A., Zenkevich E., von Borczyskowski C. (1999). Efficient low temperature charge transfer in a self-assembled porphyrin aggregate. J. Photochem. Photobiol. A: Chemistry.

[107] Nagai K., Jiang L., Iyoda T., Fujishima A., Hashimoto K. (1998). Grain size of a hard molecule-based-magnet of manganese porphyrin-tetracyanoethylene charge transfer salt. Thin Solid Films.

[108] Cai Z.-L., Crossley M.J., Reimers J.R., Kobayashi R., Amos R.D. (2006). Density Functional Theory for Charge Transfer: The Nature of the N-Bands of Porphyrins and Chlorophylls Revealed through CAM-B3LYP, CASPT2, and SAC-CI Calculations. J. Phys. Chem. B.

[109] Hasegawa J.-y., Takata K., Miyahara T., Neya S., Frisch M. J., Nakatsuji H. (2005). Excited States of Porphyrin Isomers and Porphycene Derivatives: A SAC-CI Study. J. Phys. Chem. A.

[110] Schweitzer-Stenner R., Stichternath A., Dreybrodt W., Jentzen W., Song X.-Z., Shelnutt J.A., Nielsen O.F., Medforth C.J., Smith K.M. (1997). Raman dispersion spectroscopy on the highly saddled nickel(II)-octaethyltetraphenylporphyrin reveals the symmetry of nonplanar distortions and the vibronic coupling strength of normal modes. J. Chem. Phys..

[111] Parusel A.B.J., Grimme S. (2001). DFT/MRCI calculations on the excited states of porphyrin, hydroporphyrins, tetrazaporphyrins and metalloporphyrins. J. Porphyrins Phthalocyanines.

[112] Gleiter R., Hopf H. (2004). Modern Cyclophane Chemistry.

[113] Oldham W.J., Miao Y.-J., Lachicotte R.J., Bazan G.C. (1998). Stilbenoid Dimers: Effect of Conjugation Length and Relative Chromophore Orientation. J. Am. Chem. Soc..

[114] Wang S., Bazan G.C., Tretiak S., Mukamel S. (2000). Oligophenylenevinylene Phane Dimers: Probing the Effect of Contact Site on the Optical Properties of Bichromophoric Pairs. J. Am. Chem. Soc..

[115] Sakai T., Satou T., Kaikawa T., Takimiya K., Otsubo T., Aso Y. (2005). Syntheses, Structures, Spectroscopic Properties, and π-Dimeric Interactions of [n.n]Quinquethiophenophanes. J. Am. Chem. Soc..

[116] Hong J.W., Benmansour H., Bazan G.C. (2003). Through-Space Delocalized Water-Soluble Paracyclophane Bichromophores: New Fluorescent Optical Reporters. Chem. Eur. J..

[117] Hong J.W., Gaylord B.S., Bazan G.C. (2002). Water-Soluble Oligomer Dimers Based on Paracyclophane: A New Optical Platform for Fluorescent Sensor Applications. J. Am. Chem. Soc..

[118] Williams R.V., Edwards W.D., Mitchell R.H., Robinson S.G. (2005). A DFT Study of the Thermal, Orbital Symmetry Forbidden, Cyclophanediene to Dihydropyrene Electrocyclic Reaction. Predictions to Improve the Dimethyldihydropyrene Photoswitches. J. Am. Chem. Soc..

[119] Mitchell R.H., Brkic Z., Sauro V.A., Berg D.J. (2003). A Photochromic, Electrochromic, Thermochromic Ru Complexed Benzannulene: an Organometallic Example of the Dimethyldihydropyrene-Metacyclophanediene Valence Isomerization. J. Am. Chem. Soc..

[120] Mitchell R.H., Ward T.R., Chen Y., Wang Y., Weerawarna S.A., Dibble P.W., Marsella M.J., Almutairi A., Wang Z.-Q. (2003). Synthesis and Photochromic Properties of Molecules Containing [e]-Annelated Dihydropyrenes. Two and Three Way π-Switches Based on the Dimethyldihydropyrene-Metacyclophanediene Valence Isomerization. J. Am. Chem. Soc..

[121] Williams R.V., Armantrout J.R., Twamley B., Mitchell R.H., Ward T.R., Bandyopadhyay S. (2002). A Theoretical and Experimental Scale of Aromaticity. The First Nucleus-Independent Chemical Shifts (NICS) Study of the Dimethyldihydropyrene Nucleus. J. Am. Chem. Soc..

[122] Woo H.Y., Hong J.W., Liu B., Mikhailovsky A., Korystov D., Bazan G.C. (2005). Water-Soluble [2.2]Paracyclophane Chromophores with Large Two-Photon Action Cross Sections. J. Am. Chem. Soc..

[123] Woo H.Y., Korystov D., Mikhailovsky A., Nguyen T.-Q., Bazan G.C. (2005). Two-Photon Absorption in Aqueous Micellar Solutions. J. Am. Chem. Soc..

[124] Bartholomew G.P., Rumi M., Pond S.J.K., Perry J.W., Tretiak S., Bazan G.C. (2004). Two-Photon Absorption in Three-Dimensional Chromophores Based on [2.2]-Paracyclophane. J. Am. Chem. Soc..

[125] Nelsen S.F., Konradsson A.E., Telo J.P. (2005). Pseudo-para-dinitro[2.2]paracyclophane Radical Anion, a Mixed-Valence System Poised on the Class II/Class III Borderline. J. Am. Chem. Soc..

[126] Yan X.Z., Pawlas J., Goodson T., Hartwig J.F. (2005). Polaron Delocalization in Ladder Macromolecular Systems. J. Am. Chem. Soc..

[127] Lin H.-C., Hsu J.-H., Lee C.-K., Tai O.Y.-H., Wang C.-H., Chou C.-M., Chen K.-Y., Wu Y.-L., Luh T.-Y. (2009). Observing Second-Order Nonlinear Optical Properties by Symmetry Breaking in Centrosymmetric Furan-Containing Oligoaryl Cyclophandienes. Chem. Eur. J..

[128] Hoffmann R. (1971). Cyclophane chemistry: bent and battered benzene rings. Acc. Chem. Res..

[129] Hoffmann R., Imamura A., Hehre W.J. (1968). Benzynes, dehydroconjugated molecules, and the interaction of orbitals separated by a number of intervening sigma bonds. J. Am. Chem. Soc..

[130] Pople J.A., Beveridge D.L. (1970). Approximate Molecular Orbital Theory.

